# Integrated Design of a Membrane‐Lytic Peptide‐Based Intravenous Nanotherapeutic Suppresses Triple‐Negative Breast Cancer

**DOI:** 10.1002/advs.202105506

**Published:** 2022-03-04

**Authors:** Charles H. Chen, Yu‐Han Liu, Arvin Eskandari, Jenisha Ghimire, Leon Chien‐Wei Lin, Zih‐Syun Fang, William C. Wimley, Jakob P. Ulmschneider, Kogularamanan Suntharalingam, Che‐Ming Jack Hu, Martin B. Ulmschneider

**Affiliations:** ^1^ Department of Chemistry King's College London London SE1 1DB UK; ^2^ Synthetic Biology Group Research Laboratory of Electronics Massachusetts Institute of Technology Cambridge MA 02139 USA; ^3^ Institute of Biomedical Sciences Academia Sinica Taipei 115 Taiwan; ^4^ Department of Biochemistry and Molecular Biology Tulane University New Orleans LA 70112 USA; ^5^ Department of Physics Institute of Natural Sciences Shanghai Jiao Tong University Shanghai 200240 China; ^6^ School of Chemistry University of Leicester Leicester LE1 7RH UK

**Keywords:** anticancer peptides, drug resistance, membrane‐active anticancer agents, multicellular tumor spheroids, murine models, nanoparticles, triple negative breast cancer

## Abstract

Membrane‐lytic peptides offer broad synthetic flexibilities and design potential to the arsenal of anticancer therapeutics, which can be limited by cytotoxicity to noncancerous cells and induction of drug resistance via stress‐induced mutagenesis. Despite continued research efforts on membrane‐perforating peptides for antimicrobial applications, success in anticancer peptide therapeutics remains elusive given the muted distinction between cancerous and normal cell membranes and the challenge of peptide degradation and neutralization upon intravenous delivery. Using triple‐negative breast cancer as a model, the authors report the development of a new class of anticancer peptides. Through function‐conserving mutations, the authors achieved cancer cell selective membrane perforation, with leads exhibiting a 200‐fold selectivity over non‐cancerogenic cells and superior cytotoxicity over doxorubicin against breast cancer tumorspheres. Upon continuous exposure to the anticancer peptides at growth‐arresting concentrations, cancer cells do not exhibit resistance phenotype, frequently observed under chemotherapeutic treatment. The authors further demonstrate efficient encapsulation of the anticancer peptides in 20 nm polymeric nanocarriers, which possess high tolerability and lead to effective tumor growth inhibition in a mouse model of MDA‐MB‐231 triple‐negative breast cancer. This work demonstrates a multidisciplinary approach for enabling translationally relevant membrane‐lytic peptides in oncology, opening up a vast chemical repertoire to the arms race against cancer.

## Graphical abstract

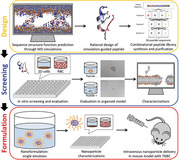

## 1 Introduction

In the search for next‐generation chemotherapeutics, membrane‐perforating peptides have been proposed as a promising treatment modality.^[^ ^1^, ^2^ ^]^ Peptides that permeabilize membranes are a ubiquitous part of the innate immune defense and have long been envisioned as therapeutic candidates against bacteria, fungi, and viruses.^[^ ^3^, ^4^, ^5^, ^6^ ^]^ Against cancer, membrane‐lytic peptides can lead to rapid necrotic cell death of their target cells,^[^ ^7^ ^]^ and the growing enthusiasm toward these tailorable therapeutic candidates are accompanied by increasingly sophisticated efforts at expanding their synthetic flexibilities and chemical repertoire.^[^ ^8^, ^9^ ^]^ The action mechanism via physical damage by membrane‐perforating peptides may help address several common shortcomings among standard‐of‐care small molecule chemotherapeutics targeting biochemical pathways, including drug‐resistance development and ineffectiveness against quiescent cancer cells that are frequently associated with treatment failure and tumor relapse.^[^ ^10^ ^]^

Continuing efforts at harnessing the potential of membrane‐perforating peptides for treating cancer have ushered in several designs and screening strategies for identifying anticancer peptides (ACPs) with improved activity.^[^ ^11^ ^]^ To overcome the translational challenges of peptide‐based drugs, including low apparent activity as compared to small‐molecule drugs and poor proteolytic stability and pharmacokinetics, a wide array of cross‐disciplinary approaches have emerged in recent literature, including the development of peptidomimetics,^[^ ^12^ ^]^ peptide‐drug conjugates,^[^ ^13^, ^14^ ^]^ stapled peptides,^[^ ^15^, ^16^ ^]^ and peptide‐based nanoparticles.^[^ ^17^ ^]^ Despite the tremendous progress made, clinical translation of ACPs still faces steep barriers, including selective targeting of the subtle distinctions between cancerous and healthy cell membranes^[^ ^18^, ^19^, ^20^, ^21^ ^]^ and enabling clinically relevant routes of administration.^[^ ^22^, ^23^ ^]^ In particular, the clinically preferred intravenous delivery route remains elusive for most of the proposed peptide and peptide‐analog chemistries as the journey between the site of injection and site of action present many biological barriers that can undermine peptide‐based therapeutics.^[^ ^24^ ^]^

**Figure 1:**
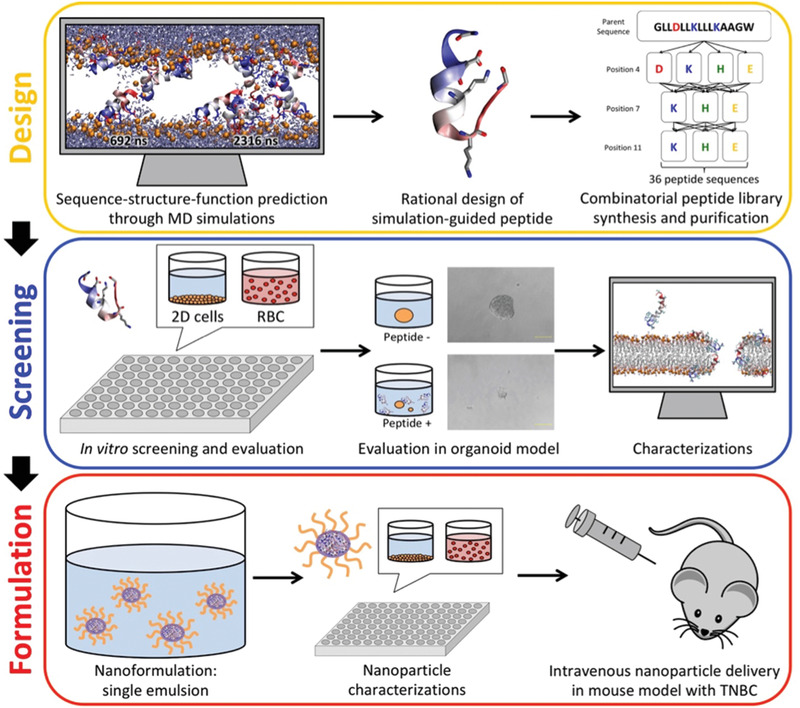
Schematic diagram of integrated design of peptide‐based nanomedicine in cancer treatment. The process of nanomedicine development involves peptide drug design, evaluation against both 2D and 3D cell cultures, and nanomedicine formulation for animal study.

Here, we demonstrate a multidisciplinary approach that integrates rational combinatorial ACP design with in silico modeling, orthogonal live‐cell activity for selectivity screening, tumor spheroid for resistance evaluation in vitro, and nanocarrier delivery for in vivo administration, to yield a membrane‐perforating ACP nanotherapeutic that is amenable to intravenous administration (**Figure** **1**). We demonstrate that the ACP nanotherapeutic is highly selective to cancerous cells, effective against multiple breast cancer cell lines and tumorspheres, resilient against the drug‐resistance formation, and effective against xenografts of human triple‐negative breast cancer (TNBC), which is notorious for having limited treatment options.^[^ ^25^ ^]^

To develop the ACP we followed a rational design approach as summarized in Figure S1, Supporting Information. Cancer cell selectivity was programmed into a generic pore‐forming sequence by modulating the charge distribution. Peptide candidates with high anticancer activity and cancer cell selectivity were simultaneously identified by in vitro screening against several tumor and non‐tumor cell lines. Lead peptides were further evaluated in multicellular spheroids. Biophysical experiments of peptide‐lipid interactions, in vitro assessment of cell death pathway, and all‐atom molecular dynamics simulations of peptide self‐assembly and peptide‐induced membrane permeabilization were applied to gain mechanistic insights into the molecular mechanisms of perforation. To address the peptides’ proteolytic susceptibility and plasma neutralization upon intravenous administration,^[^ ^5^, ^16^, ^26^, ^27^ ^]^ an optimized nanoprecipitation method was adopted to encapsulate the membrane‐lytic peptides in ultrasmall, 20 nm polymeric nanocarriers.^[^ ^28^, ^29^ ^]^ Comprised of an acid‐labile, a biodegradable polymer with a diminutive dimension, the peptide nanoparticles exhibit a desirable release profile critical for the perforating function of the encapsulated peptides. By combining rational peptide design, in vitro screening, molecular modeling, and intravenous nanoparticle delivery, this study demonstrates an integrated approach for developing clinically viable peptide‐based therapeutics for cancer treatment.

## 2 Results

### 2.1 Peptide Design

Membrane‐perforating peptides produced by many living organisms have provided principles for synthetic peptide designs through molecular simulation.^[^ ^4^ ^]^ Using simulations to probe these principles and guide peptide design can produce peptides that perforate membranes at very low peptide‐to‐lipid ratios (<1:1000).^[^ ^40^ ^]^ Here we attempt to re‐engineer peptides to be selectively active toward cancerous cells by 1) tuning the peptide pKa and charge distribution, 2) rearranging the charged amino acids to modulate electrostatic interactions with cancer cell membrane lipids, while 3) maintaining peptide hydrophobicity that we speculated to be essential for peptide pore formation.

Hence, we designed a 36‐member ACP library: GLLxLLxLLLxAAGW, where x may be D, E, H, or K (Table S1, Supporting Information). This library enables targeting the optimization of the acidic tumor microenvironment (pH = 6.5–6.8, compared to 7.2–7.5 for healthy tissues)^[^ ^41^ ^]^ through modification of the net template charge (−2 to +4) while maintaining the hydrophobic moment essential for pore formation (Figure S1, Supporting Information).

### 2.2 Cancer Cell Activity and Selectivity

**Figure 2:**
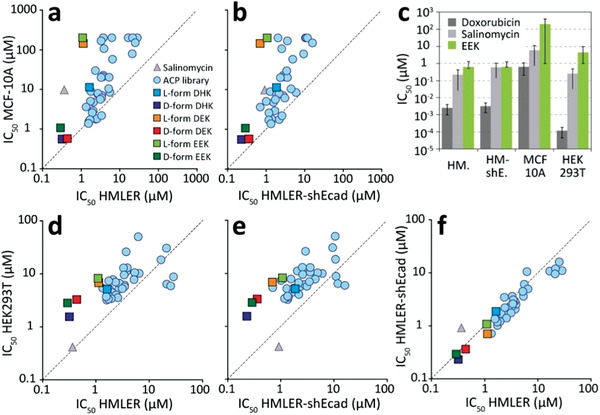
Compounds selectivity for cancer cells in vitro. a) Comparison of compound IC_50_ against cancerous (HMLER) and noncancerous (MCF‐10A) human breast epithelial cells in a 2D in vitro culture model. b) Comparison of IC_50_ epithelial breast cancer stem cell (HMLER‐shEcad) and noncancerous MCF‐10A. c) Comparison of IC_50_ values for doxorubicin, salinomycin, and EEK against HMLER, HMLER‐shEcad, MCF‐10A, and HEK293T. d) IC_50_ comparison of HMLER and HEK293T. e) IC_50_ comparison of HMLER‐shEcad and HEK293T. f) Comparison of selectivity for HMLER and HMLER‐shEcad.

In vitro dose‐response cytotoxicity screening in a 2D culture model (Figure S2, Supporting Information) revealed that all ACPs are active against both human breast epithelial cancer cells (HMLER) and human breast epithelial cancer stem cells (HMLER‐shEcad), with an average IC_50_ of 4.8 ± 5.8 µm (range: 1–25 µm) (**Figure** **2**f and Table S2, Supporting Information). Comparison with activity against non‐tumorigenic breast endothelial cells (MCF‐10A), which serves as a normal cell control, revealed six library peptides (EEK, DEK, KEE, EEH, EEE, and EHE) with >25‐fold selectivity for cancerous cells (Figure 2a,b), the most significant being DEK (>120‐fold) and EEK (>180‐fold) (Figure 2c). ACPs are slightly more toxic to human embryonic kidney cells (HEK293T; an immortalized cell line) than non‐tumorigenic MCF‐10A breast epithelial cells and retain their significant selectivity for cancer cells (Figure 2c–e). D‐enantiomeric ACPs have even higher anticancer activity at sub‐nanomolar concentrations but have poor cancer cell selectivity (Figure S3, Supporting Information).

Comparison with the FDA‐approved anticancer agent salinomycin (FDA ODD: HSB‐1216), an apoptosis‐inducing ionophore that has been shown to be effective at targeting breast cancer stem cells,^[^ ^42^ ^]^ revealed that ACPs have similar activity, but superior selectivity toward breast cancer cells (Figure S3, Supporting Information). Doxorubicin has 1000‐fold stronger anticancer activity (IC_50_ = 1.6 nm) than the ACPs and >160‐fold selectivity for cancerous over healthy breast endothelial cells. However, both doxorubicin and salinomycin are more toxic to HEK293T than cancer cells (Figure 2c).

### 2.3 Activity against Multicellular Spheroids

**Figure 3:**
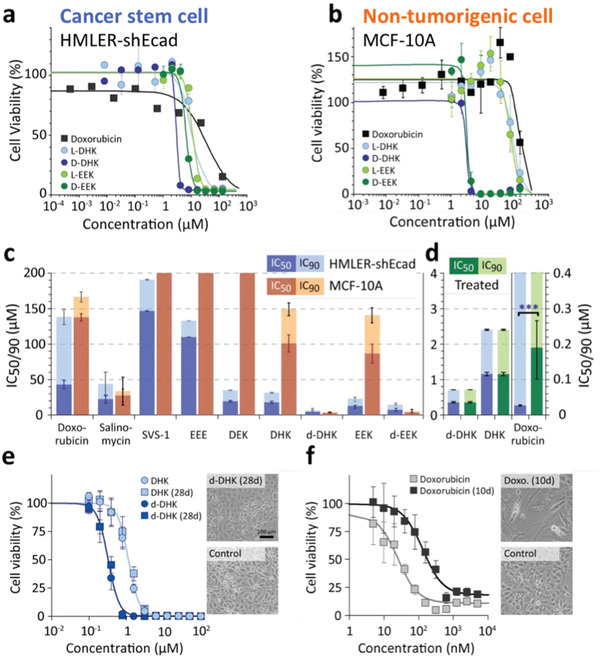
Comparison of compound activity against cancer stem cell spheroids and evaluation of resistance formation. a) Dose‐dependent cell viability of HMLER‐shEcad (cancer stem cell) tumorspheres treated with either doxorubicin, DHK, EEK, or the two ACP D‐enantiomers d‐DHK and d‐EEK. b) Dose‐dependent cell viability of MCF‐10A (non‐tumorigenic cell) mammospheres treated with the same compounds. c) IC_50_ (dark color) and IC_90_ (light color) of all compounds tested against HMLER‐shEcad tumorspheres (blue) and MCF‐10A mammospheres (red). d) IC_50_ (dark color) and IC_90_ (light color) of doxorubicin, DHK, and d‐DHK, in 2D HMLER‐shEcad cells after prolonged exposure to vehicle (blue) and doxorubicin or d‐DHK (green). e) Dose‐dependent cell viability of 2D HMLER‐shEcad after 28 days of co‐incubation with 0.4 µm d‐DHK or vehicle. The scale bar is 100 µm. f) Dose‐dependent cell viability of 2D HMLER‐shEcad cells after 10 days of co‐incubation with either 30 nm doxorubicin or vehicle.

We next evaluated the anti‐tumor activity and cancer cell selectivity in a 3D multicellular spheroid mammary tissue model, which is more representative of in vivo tumors.^[^ ^43^ ^]^ Dose‐response measurements revealed that DHK, DEK, EEK, and the two D‐enantiomers d‐DHK, d‐EEK have superior activity against HMLER‐shEcad tumorspheres (average IC_50_ = 12 ± 2 µm) compared to both doxorubicin (IC_50_ = 43 ± 6 µM) and salinomycin (IC_50_ = 22 ± 5 µm), and the previously reported membrane‐lytic anticancer peptide SVS‐1^11^ (IC_50_ = 147 ± 1 µm) (**Figure** **3**a and Figure S4a, Supporting Information).

Concentration‐dependent cell viability shows a steep sigmoidal decline to <6% for all six ACPs tested (Figure 3a). In contrast, doxorubicin shows a more gradual decline in cell viability, with >15% viability even at 133 µm (Figure 3a). Such dramatic reduction in chemotherapy effectiveness against tumorspheres is previously reported,^[^ ^44^ ^]^ and it can be attributed to the increased fraction of quiescent cells in tumorspheres compared to 2D cell culture models.^[^ ^43^ ^]^ Unlike doxorubicin, ACPs are able to effectively target quiescent cells. Reducing tumorsphere cell viability by 90% requires significantly higher concentrations of both doxorubicin (IC_90_ = 138 ± 11 µm) and salinomycin (IC_90_ = 44 ± 17 µm) compared to the five active ACPs (DEK, DHK, d‐DHK, EEK, and d‐EEK; average IC_90_ = 22 ± 2 µm) (Figure 3c and Figure S4a, Supporting Information).

Comparison with MCF‐10A mammospheres reveals that L‐form ACPs show significant selectivity, while the two D‐enantiomers (d‐DHK, d‐EEK), doxorubicin, and salinomycin lack cancer cell selectivity, exhibiting similar toxicities against the mammospheres (D‐enantiomer average IC_90_ = 8 ± 5 µm; doxorubicin: IC_90_ = 167 ± 7 µm; salinomycin: IC_90_ = 34 ± 20 µm) as to the tumorspheres (Figure 3b and Figure S4b, Supporting Information). Of the compounds tested, EEK has the best overall characteristics, with good activity against tumorspheres (IC_90_ = 23 ± 3 µm) and low toxicity toward mammospheres (IC_90_ = 141 ± 11 µm) (Figure 3c and Figures S4 and S5, Supporting Information).

### 2.4 Evaluating Cancer Cell Drug Resistance

Cancer stem cells can survive treatment, gain resistance, and reseed tumors, presenting a major challenge for chemotherapy.^[^ ^2^ ^]^ To evaluate resistance formation toward ACPs, we challenged HMLER‐shEcad cells with IC_50_ concentrations of either doxorubicin (30 nm) or ACP (0.4 µm d‐DHK) for up to 4 weeks. Drugs were added with medium every 3–4 days, maintaining smooth cell growth, and 125 000 cells were split into new T25 flasks with a total volume of 5 mL once per week.

After 10 days, doxorubicin‐treated HMLER‐shEcad showed significant changes in morphology and an eightfold reduction in doxorubicin sensitivity (IC_50_ increased from 24 ± 2 to 189 ± 68 nm) (Figure 3f). In contrast, after 28 days of prolonged exposure to ACPs, cells show no morphological changes and no shift in IC_50_ to either DHK or d‐DHK, indicating ACPs can be less susceptible to cancer drug resistance mutations compared to chemotherapeutics (Figure 3e).

### 2.5 Mechanism of Selectivity

To explore the molecular mechanism underpinning selectivity for cancer cells, we first evaluated ACP binding to liposomes that are either neutral (POPC) or enriched in anionic lipids (POPC:POPG = 3:1). Enrichment of plasma membranes with anionic lipids such as phosphatidylserine and sialic acid have been reported for cancer cells, including the MCF‐7 cell line, which is similar to HMLER.^[^ ^18^, ^19^, ^20^, ^45^, ^46^ ^]^ Naturally derived antimicrobial peptides that have been repurposed as ACPs are typically cationic,^[^ ^11^, ^19^, ^20^, ^46^, ^47^, ^48^ ^]^ and are thought to target cancer cell membranes enriched in anionic lipids. However, the six ACPs (EEK, DEK, KEE, EEH, EEE, and EHE) with the strongest activity against 2D HMLER cultures (IC_50_ < 10 µM) and least toxicity toward MCF‐10A cells (IC_50_ > 100 µM), are either net neutral or anionic at physiological pH. This observation suggests that the identified peptides may owe their selectivity to pH differential between cancer and normal cell surroundings.^[^ ^49^ ^]^ As cancer cells are known to produce more acidic products through reprogrammed energy metabolism, their surroundings may lead to peptide protonation states that favor membrane lytic actions by the ACPs.

Further examination by tryptophan fluorescence spectroscopy measurements at physiological pH = 7.4 revealed no correlation between ACP cancer cell selectivity and binding to anionic liposomes (Table S3 and Figure S6, Supporting Information). Furthermore, ACP‐induced ANTS/DPX dye leakage is similar for both neutral and anionic liposomes at physiological pH (Table S4 and Figure S7, Supporting Information). At pH = 4.8, which mimics the acidic tumor microenvironment, 12 ACPs, including four (EEK, DEK, EHE, and EEH) of the six most cancer cell‐selective peptides have significantly increased dye leakage from anionic liposomes. However, two strongly selective ACPs (EEE and KEE) show no increase in selectivity at low pH and two cancer‐selective peptides (EHH and HEH) show increased selectivity for neutral bilayers. These analyses suggest that ACP selectivity for cancer cells is rooted in a combination of both plasma membrane lipid composition and extracellular pH.

### 2.6 Mechanism of Activity

We next explored the mechanism of anticancer activity for the two most potent cancer‐selective ACPs: DHK and EEK (IC_50_ = 1.0 ± 0.2 µm), and their non‐selective D‐enantiomers: d‐DHK and d‐EEK (IC_50_ = 0.3 ± 0.1 µm). DHK, d‐DHK, and d‐EEK are haemolytic (HC_50_ = 14 ± 5 µm), while EEK causes only minimal human red blood cell lysis below 90 µm (Figure S8a, Supporting Information). Cell viability of HMLER‐shEcad cells treated with both enantiomers of EEK cannot be rescued by co‐incubation with 20 µm of necroptosis inhibitor necrostatin‐1, nor by co‐incubation with 5 µm of the apoptosis inhibitor z‐VAD‐FMK (Figure S8b, Supporting Information), suggesting the ACP‐induced cell death is neither caused by caspase‐dependent apoptosis nor necroptosis. In contrast, the viability of HMLER‐shEcad cells treated with doxorubicin can be dramatically improved by co‐incubation with either z‐VAD‐FMK or necrostatin (Figure S8c, Supporting Information). These results suggest physical cell damage as the primary mechanism of ACP anticancer activity.

### 2.7 Anticancer Peptide Pore Structures and Function

**Figure 4:**
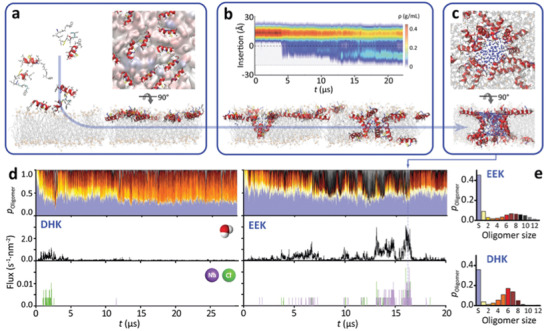
ACP membrane pore structures and membrane perforation mechanism. Molecular dynamics simulations reveal the full atomic details of a) spontaneous ACP membrane adsorption, b) insertion, and c) pore formation (shown is a large, heterogeneous, fully water‐filled EEK pore). d,e) Bound peptides form an ensemble of transient pores of 2–16 peptides (top) that conduct both water (middle) and ions (bottom) across the membrane.

Membrane‐perforating peptides typically form transient pores that elude experimental determination with current technology.^[^ ^37^ ^]^ To reveal the molecular mechanisms underpinning membrane perforation we studied folding‐partitioning and pore assembly of DHK and EEK using unbiased long‐timescale atomic detail molecular dynamics simulations.^[^ ^50^ ^]^ Both ACPs rapidly absorb and fold onto the membrane interface (**Figure** **4**a), consistent with tryptophan fluorescence and circular dichroism spectroscopy measurements that reveal strong binding of DHK to both zwitterionic POPC and anionic POPG vesicles (*∆G*_binding_ = −8.8 ± 1.2 kcal mol^−1^) (Figure S9, Supporting Information). Subsequently, on timescales of tens of µs, ACPs cooperatively insert and translocate across the lipid bilayer, populating both membrane interfaces (Figure 4b), and form an ensemble of pores (Figure 4d). Structure analysis reveals highly heterogeneous pore architectures, with the majority made up of six to ten peptides, that continuously form and disband in the membrane (surface aligned = blue, other colors correspond to oligomer size) (Figure 4e). A key structural motif is the double‐stacking of helices to span the hydrophobic core of the membrane (Figure 4c). Pores conduct both water and ions (Figure 4d), and leakage is dominated by larger more stable pores consisting of 10–12 peptides that form large aqueous channels lined with polar and charged side chains (Figure 4c).

### 2.8 Anticancer Peptide's Potential as Chemotherapy Enhancers

To explore the potential of membrane‐perforating ACPs for enhancing cellular uptake of small molecule chemotherapeutics, we co‐administered salinomycin (MW = 751 Da) with 0, 1.25, 2.5, and 5 µm DHK, respectively. At 2.5 µm DHK the dosage of salinomycin required to kill both HMLER and HMLER‐shEcad cells is reduced by a factor of 8–27 (HMLER: from IC_50_ 0.4 ± 0.1 µm to 0.05 ± 0 µm; HMLER‐shEcad: from IC_50_ 0.9 ± 0.3 µm to 0.03 ± 0 µm), while the toxicity to noncancerous MCF‐10A cells is increased by a factor of 3–6 (from IC_50_ = 9.7 ± 2.3 µm to 1.7 ± 0 µm) and HEK293T cells by a factor of 4 (from IC_50_ = 0.4 ± 0.1 µm to IC_50_ = 0.1 ± 0 µm) (Figure S10, Supporting Information). Nevertheless, the overall selectivity of the peptide‐drug combination toward cancerous cells is significantly improved (IC_50_ = 0.03–0.05 µM) while showing a 3‐ to 50‐fold reduction in toxicity to noncancerous cells (IC_50_ = 0.1–1.7 µm). The addition of 5 µm DHK results in a 100‐fold increase in anticancer efficiency, with <1% cancer cell viability at the lowest dose of salinomycin, while simultaneously increasing chemotherapeutic selectivity and reducing cytotoxicity to noncancerous cells.^[^ ^51^ ^]^

### 2.9 Nanocarrier Design and In Vivo Activity

To enable intravenous ACP delivery and address the solubility, pharmacokinetics, and stability issues that impede the translation of lytic peptide‐based therapeutics,^[^ ^5^, ^27^, ^52^ ^]^ we prepared ACP‐loaded PEG‐PLGA nanoparticles (NPs), 20 nm in diameter, using an optimized nanoprecipitation method. These ultrasmall nanocarriers have shown advantages over larger carriers in their enhanced ability to extravasate into and diffuse within tumors.^[^ ^28^, ^53^, ^54^ ^]^ In addition, the expanded overall surface area associated with the diminutive dimension of the 20 nm carriers facilitates increased water contact and enables accelerated hydrolysis‐mediated release profile suitable for the membrane‐lytic peptide therapeutic as the peptides need to regain their molecular freedom for membrane perforating actions. L‐EEK was selected for NP preparation (L‐EEK‐NPs) in the present study based on its highest cancer‐specific selectivity.

**Figure 5:**
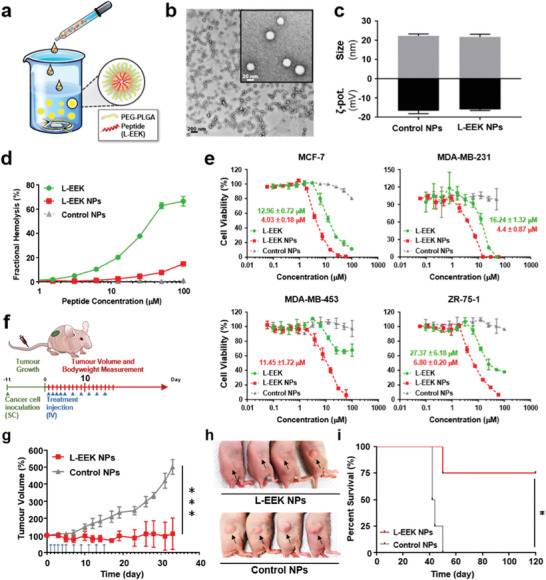
Development of EEK nanoparticles. a) Schematic illustration of L‐EEK NPs and NPs preparation. b) Transmission electron microscopy images of L‐EEK NPs. Scale bars are 200 nm (black) and 20 nm in the inset (white). c) Dynamic light scattering characterizations of L‐EEK NPs (*n* = 3) and control NPs without L‐EEK cargo. d) The comparative haemolytic activities of free L‐form EEK, L‐EEK NPs, and control NPs. e) Assessment of cell viability by CCK‐8 assay with L‐EEK, L‐EEK NPs, and control NPs treatment against breast cancer cell lines (MCF‐7, MDA‐MB‐231, MDA‐MB‐453, and ZR‐75‐1). IC_50_ values of L‐EEK and L‐EEK NPs against breast cancer cells are in green and red, respectively. f) Schematics of the mouse model of MDA‐MB‐231 triple‐negative breast cancer with control nanoparticles and EEK peptide nanoparticles treatment schedule. g) Efficient inhibition of cancer growth with L‐form EEK NPs treatment. Upon establishment of palpable tumors on day 11 following subcutaneous inoculation with MDA‐MB‐231 (4 × 10^6^ cells), mice were treated with 10 mg kg^−1^ per dose of EEK‐NPs or equivalent doses of control NPs over a 14‐day treatment period. Tumor volumes were monitored. ***p < 0.005 (*n* = 4). h) Images of MDA‐MB‐231 tumors on day 33 after the onset of L‐EEK NPs and control NPs treatments. i) Kaplan–Meier curve of mice survival following tumor inoculation over an observation period of 120 days. Mouse survival is defined as tumor size below 1000 mm^3^ (*n* = 4, *p < 0.05).

Following optimization of the nanoprecipitation protocol (**Figure** **5**a), unimodal NPs 21.7 ± 1.4 nm in diameter and with a zeta potential of −16.0 ± 0.6 mV were readily formed (Figure 5b,c). Control NPs without EEK cargo showed similar physicochemical properties (Figure S11a, Supporting Information), suggesting EEK loading is mediated via encapsulation inside the polymeric core rather than surface absorption. High‐performance liquid chromatography (HPLC) analysis of EEK‐NPs showed a high encapsulation efficiency of EEK at 82.3 ± 3.4% (Figure S11b,c, Supporting Information), translating to a peptide loading yield of 16.4 µg per mg of polymer. L‐EEK‐NPs peptide release kinetics are pH‐sensitive, relinquishing 95.3% of peptides at pH 5.0 in 4 h and ≈90% of the peptide content after 96 h at the physiological pH of 7.4 (Figure S11d, Supporting Information). Such release profile enables sustained ACP release in the tumor microenvironment and burst peptide release in the acidic endolysosomal environment upon cancer cell uptake. Using fluorescently labeled L‐EEK peptides, we further show that NP encapsulation increased intracellular peptide delivery in four different breast cancer cell lines (MCF‐7, MDA‐MB‐231, MDA‐MB‐453, and ZR‐75‐1) (Figure S11e, Supporting Information). Cell viability assessed by CCK‐8 assay with control NPs, L‐EEK peptides, and L‐EEK‐NPs treatment further showed that the NP formulation increased EEK anticancer efficacy by a factor of 4 against the four different breast cancer cell lines (Figure 5e), suggesting that the ACPs may retain its cytolytic function within the endosomal environment where the invaginated membrane composition is largely similar to the plasma membrane.^[^ ^55^ ^]^ While enhancing peptide cytotoxicity against cancer cells, NPs were further shown to reduce peptide interaction with red blood cells (Figure 5d).

The therapeutic relevance of L‐EEK‐NPs was assessed in a mouse model of MDA‐MB‐231 breast cancer (Figure 5f), which is a TNBC known for having a poor prognosis and limited treatment options. Upon establishment of palpable tumors, mice were treated with either control NPs or L‐EEK‐NPs over a 2‐week treatment course. Upon tumor observation following the treatment period, mice in the control group exhibited significant tumor growth in volume (Figure 5g,i and Figure S12a, Supporting Information). In contrast, mice that received EEK‐NP treatment showed significantly inhibited tumor growth (Figure 5g,i and Figure S12b, Supporting Information), with two of the four treated mice showing complete tumor eradication. Notably, both control NP and L‐EEK‐NP treatments showed negligible body weight loss (Figure 5h and Figure S12c,d, Supporting Information), attesting to the safety of the ACP nanoformulation.

Prior studies on membrane lytic peptides for cancer treatment often see a reduction in peptide activities upon interaction with serum proteins and blood cells, thereby dampening their translational potential.^[^ ^56^, ^57^ ^]^ The prominent anticancer efficacy by the intravenous L‐EEK‐NP treatment demonstrates in vivo retention of selective membrane‐lytic peptide activity upon integration with properly designed delivery strategies, opening up vast opportunities in therapeutic designs afforded by the synthetic flexibility of synthetic peptides.

## 3 Discussion

### 3.1 Anticancer Peptide Development

Several membrane‐perforating peptides are clinically approved as antibiotics,^[^ ^5^ ^]^ highlighting the translational potential toward expanding this compound class for cancer therapy. Anticancer activity has been reported for a number of membrane‐perforating peptides, typically derived from cationic antimicrobial peptides.^[^ ^58^ ^]^ Despite the strong activity of these naturally occurring peptides, cancer selectivity over normal cells is typically muted.^[^ ^59^, ^60^ ^]^ Efforts at improving selectivity for cancer cells have focused on introducing and varying the number and spacing of cationic residues,^[^ ^59^, ^61^, ^62^ ^]^ in order to target the negative surface charge of malignant cells that are enriched in anionic lipids like phosphatidylserine.^[^ ^11^, ^18^, ^19^, ^20^, ^46^, ^47^ ^]^

A number of cationic ACPs have been evaluated preclinically (**Table** **1**), and at least one peptide (LTX‐315) is currently in phase I/II clinical trials (ClinicalTrials.gov; National Clinical Trials number: NCT03725605, NCT04796194, NCT01223209, NCT01986426, and NCT01058616).^[^ ^22^, ^63^ ^]^ In contrast, anionic and neutral peptides are unusual, as their interaction with anionic cancer cell membranes is difficult to rationalize. Our goal here was to explore neutral (i.e., zwitterionic) and anionic sequences in order to improve targeting of the subtle changes in lipid composition and surface‐pH of malignant cell plasma membranes. We hypothesize that cancer membrane selectivity of membrane‐lytic peptides may be enhanced with the purposeful introduction of anionic residues,^[^ ^14^, ^64^, ^65^ ^]^ which remain a largely unexplored targeting modality among ACPs. The deliberate addition of anionic amino acids was also aimed at addressing the in vivo delivery challenges of overly cationic peptides,^[^ ^9^, ^66^ ^]^ which have been associated with poor pharmacokinetics and toxicity concerns.

**Table 1:** ACP activity comparison. Reported in vitro anticancer activity (IC_50_) of ACPs

ACP	IC_50_ [µm]	Cancer cell	Assay and Notes	References
Magainin II	45	A549	2D and mouse xenograft model (dose: 25 mg kg^−1^); The tumor in mouse was smaller and still growing in 40 days	^[^ ^68^ ^]^
LL37	40	HCT116	2D; Vascular toxicity	^[^ ^69^ ^]^
Aurein 1.2	10–100	various	2D (no data for cytotoxicity and haemolysis)	^[^ ^70^ ^]^
Cecropins	10–100	various	2D and mouse xenograft model (dose: unknown mg kg^−1^); The tumor in mouse was smaller and still growing in 35 days	^[^ ^71^ ^]^
LTX‐315	8–34	A20, AT84, MRC‐5	In phase I/II	^[^ ^22^, ^23^, ^57^, ^63^ ^]^ and NCT03725605, NCT04796194, NCT01223209, NCT01986426, NCT01058616
L‐K6	23	MCF‐7	2D and mouse xenograft model (dose: 10 mg kg^−1^); The tumor in mouse was smaller and still growing in 15 days	^[^ ^20^ ^]^
Decoralin	13	MCF‐7	2D; haemolytic	^[^ ^72^ ^]^
SVS‐1	11	MCF‐7	2D; in vitro drug resistance was observed	^[^ ^11^, ^19^, ^47^ ^]^
EEK	1–27 (13)	various (MCF‐7)	2D and 3D tumorspheres	Figures 2, 3, and 5
EEK‐NP	4 4–12	MCF‐7 MDA‐MB‐231, MDA‐MB‐453, ZR‐75‐1	2D and mouse xenograft model (dose: 10 mg kg^−1^)	Figure 5

To develop our ACP library we varied acid strength, net charge, and charge distribution along the sequence (Figure S1, Supporting Information) of a non‐selective pore‐forming template sequence.^[^ ^40^ ^]^ Remarkably, our lead ACP (EEK) is neutral (taking the positive N‐terminus into account) and several active ACPs in the library (e.g., EEE) are anionic, underscoring the unpredictability and complexity of tuning cancer selectivity. It has been noted before that the mutations often produce counter‐intuitive effects,^[^ ^67^ ^]^ highlighting that sequence‐activity relationships are non‐trivial and ACP optimization will remain challenging.

### 3.2 In Vitro Anticancer Peptide Activity

IC_50_ determined from 2D in vitro assays allows for rapid screening and evaluation of ACPs. ACPs evaluated using this method typically have IC_50_ between 10–100 µm (Table 1). In these assays, established small molecule drugs outperform all known ACPs, including EEK, by orders of magnitude (Figure 2c). Our data shows that IC_90_ may be a better indicator of therapeutic efficacy, as it more closely reflects the ability of a compound to eradicate all cancer cells. For example, the IC_50_ of doxorubicin (Figure 3d,f) is >100‐fold lower than the best ACPs in this study (Figure 3d,e). However, the IC_90_ of doxorubicin could not be attained as a significant number of cells remained viable with the drug in the high micromolar range. On the other hand, the ACPs killed all cancer cells at low micromolar concentrations.

Recent advances in 3D spheroid cell culture models allow capturing of the biological complexities of the tumor microenvironment.^[^ ^73^ ^]^ Specifically, spheroids consist of cohorts of cancer cells at various cell‐cycle stages, including quiescent and oxygen‐deprived cells. These form naturally in spheroids and capture many features of real tumors,^[^ ^43^ ^]^ providing a better indication of in vivo drug performance. Remarkably, in a spheroid assay SVS‐1 shows similar performance to doxorubicin, while the ACP family presented here shows superior performance compared to doxorubicin, salinomycin, and SVS‐1 (Figure 3a–c and Figure S4, Supporting Information), while in 2D assays doxorubicin and salinomycin outperformed the ACPs by two to three orders of magnitude (Figure 2a–c). Furthermore, the large therapeutic window (>100‐fold IC_50_) observed in 2D assays for doxorubicin narrows dramatically in the 3D assay (≈3‐fold IC_50_). Together these data suggest that 2D assays may bias against ACPs compared to small molecules as performance indicators. However, 2D assays remain useful for measuring resistance formation, with current work confirming no detectable resistance formation of ACPs (Figure 3e).

### 3.3 Mechanism of Activity

A key impediment to the translation of ACPs has been the difficulty in capturing the molecular mechanisms underpinning membrane‐perforation activity. Here we show that this barrier is being eroded due to the tremendous advances in unbiased MD simulations that now offer a route to reveal these dynamic processes in fluid lipid bilayers at atomic resolution.^[^ ^50^, ^62^, ^65^, ^74^ ^]^ The information obtained from these simulations can now provide detailed quantitative information on peptide‐lipid interactions that favor membrane perforation (Figure 4).

### 3.4 Translational Considerations: Stability, Pharmacokinetics, and Delivery Options

Low plasma stability, poor pharmacokinetics, and limited delivery options remain major obstacles to the translation of ACPs. Peptide stability can be addressed in a variety of ways, such as using D‐enantiomers or backbone cyclisation to resist proteolytic enzymes. Our ACPs retain potent activity as D‐forms, however, selectivity is lost, highlighting that stabilizing modifications may alter pharmaceutical properties.

Recent years have seen tremendous progress with nanoformulated delivery vehicles, in particular polymeric NPs.^[^ ^54^, ^75^ ^]^ Integration of the delivery technology helps address the translational barriers of peptide therapeutics, opening the door for previously inaccessible peptide chemistries, including ACPs, into the clinic. We show that a biocompatible NP formulation of our lead ACP (EEK) is intravenously deliverable, shows little haemolytic activity (Figure 5d), and possesses enhanced anticancer activity over free peptide (Fig 5e). The higher in vitro activity of the nanoformulation may be attributed to the delivery of ACPs as a concentrated burst on a localized patch of the target membrane by the nanocarrier, preventing peptide dilution and precipitation.

## 4 Conclusion 

Membrane‐perforating peptides present a vast pharmacological reservoir for the development of highly effective targeted anticancer treatments with low peripheral toxicity. This study demonstrates that seamless integration of multiple emerging techniques, ranging from 3D cell culture models, atomic detail molecular simulations, and nanotechnology enables development, tuning, characterization, and demonstration of clinically relevant delivery of cancer‐selective ACPs that kill breast cancer cells at nontoxic levels, reveals a remarkable resilience against drug resistance formation, and can inhibit growth or eradicate human TNBC xenografts in mice. Building on an enormous body of work in the field of ACPs, this study shows that clinical translation of membrane‐perforating peptides may finally be within reach. The need for new treatment modalities has never been more pressing, as even the recently approved TNBC treatment, sacituzumab govitecan, prolongs life (median overall survival) by an average of 12 months, compared to 7 months for chemotherapy.^[^ ^76^ ^]^

## 5 Experimental Section

### Reagents and Materials

Peptides (>95% purity) were synthesized using solid‐phase peptide synthesis, purified using HPLC with C18 column, verified by ESI mass spectrometry and LC‐MS, and purchased from GenScript (Piscataway, NJ). Doxorubicin hydrochloride, salinomycin, necrostatin‐1, thiazolyl blue tetrazolium bromide (MTT), in vitro toxicology assay kit (TOX8), and SYTOX green nucleic acid stain were purchased from Sigma‐Aldrich (St. Louis, MO). Z‐VAD‐FMK was purchased from Selleck Chemicals (Houston, TX). Cell Counting Kit‐8 (CCK‐8) was purchased from Dojindo Molecular Technologies (Rockville, MD). Trifluoroacetic acid (TFA) was purchased from Alfa Aesar (Heysham, England). Acetonitrile and methanol were purchased from J. T. Baker (Avantor Performance Materials, Center Valley, PA). Mammalian cell lines HeLa, U2OS, HEK293T, MCF‐10A, and their media mammary epithelial cell growth medium (MEGM) and Dulbecco's Modification of Eagle's Medium (DMEM) were purchased from ATCC (Manassas, VA). MCF‐7, MDA‐MB‐231, MDA‐MB‐453, and ZR‐75‐1 were purchased from Bioresource Collection and Research Center (BCRC, Taiwan). The human mammary epithelial cell lines, HMLER and HMLER‐shEcad, were kindly donated by Prof. R. A. Weinberg (Whitehead Institute, MIT). Fresh human red blood cells were obtained from Interstate Blood Bank (Philadelphia, PA). 8‐aminonaphthalene‐1,3,6‐trisulfonic acid, disodium salt (ANTS), and *p*‐xylene‐bis‐pyridinium bromide (DPX) were purchased from Thermo Fisher Scientific (Waltham, MA). Synthetic lipids and Avanti Mini Extruder were purchased from Avanti Polar Lipids (Alabaster, AL). Poly(ethylene glycol) methyl ether‐*block*‐poly(lactide‐*co*‐glycolide) (PEG‐PLGA; PEG average *M*_n_ = 5000, PLGA *M*_n_ = 7000) was purchased from Sigma‐Aldrich (St. Louis, MO). Sodium pyruvate, DMEM, glutamine, and culture multi‐well plates were purchased from Corning (Corning, NY). All reagents and chemicals were analytical grade and purchased from Fischer Scientific (Hampton, NH).

### Cell Lines and Cell Culture Conditions

HMLER, HMLER‐shEcad, and MCF‐10A cells were maintained in MEGM with supplements and growth factors bovine pituitary extract, hydrocortisone, human epidermal growth factor, insulin, and gentamicin/amphotericin‐B. U2OS and HEK293T cells were maintained in DMEM with a final concentration of 10% fetal bovine serum (FBS). MDA‐MB‐231 and MDA‐MB‐453 were grown in 90% Leibovitz's L‐15 medium supplemented with 2 mm glutamine, 10% FBS, and 1% penicillin/streptomycin. MCF‐7 cells were maintained in DMEM containing 5% FBS and 1% penicillin/streptomycin. ZR‐75‐1 were cultured in 90% RPMI 1640 medium with 2 mm L‐glutamine adjusted to contain 1.5 g L^−1^ sodium bicarbonate, 4.5 g L^−1^ glucose, 10 mm HEPES, 1.0 mm sodium pyruvate, 10% FBS, and 1% penicillin/streptomycin. Cells were grown in T75 flasks at 37 °C in a humidified atmosphere containing 5% CO_2_.

### Cytotoxicity Assay

The colorimetric MTT (3‐(4,5‐dimethylthiazol‐2‐yl)‐2,5‐diphenyltetrazolium bromide) assay was used to determine the cytotoxicity of ACPs and anticancer drugs. 5 × 10^3^ cells were seeded in each well of 96‐well microplates and incubated overnight. Serial dilutions of the compounds (0, 0.1, 0.2, 0.4, 0.8, 1.6, 3.1, 6.3, 12.5, 25, 50, and 100 µm) were added and incubated for another 72 h with a total volume of 200 µL. The compound stock was prepared as 5 mm solutions either in DMSO or pure water, and the stock solution was diluted using DPBS buffer. The final concentration of DMSO in each well was either 0.5% or 0% and this amount was present in the untreated control. After 72 h incubation, 20 µL of a 4 mg mL^−1^ solution of MTT in phosphate‐buffered saline (PBS) was added to each well, and the plate was incubated for an additional 4 h. The MEGM/MTT mixture was aspirated and 100 µL of DMSO was added to dissolve the resulting purple formazan crystals. The absorbance of the solutions in each well was read at 550 nm. Absorbance values were normalized to either DMSO‐containing or no DMSO‐containing control wells and plotted as the concentration of test compound versus % cell viability. IC_50_ values were interpolated from the resulting dose‐dependent curves. The reported IC_50_ values were the average of two independent experiments, each consisting of six replicates per concentration level (overall *n* = 12). The IC_50_ values for 36 leucine‐rich peptides were the average of two independent experiments (overall *n* = 2).

### Tumorsphere Formation and Viability Assay

HMLER‐shEcad cells (5 × 10^3^) were plated in ultralow‐attachment 96‐well plates (Corning) and incubated in MEGM supplemented with B27 (Invitrogen), 20 ng mL^−1^ EGF, and 4 µg mL^−1^ heparin (Sigma) for 5 days. Studies were conducted in the absence and presence of ACPs, doxorubicin, and salinomycin. Mammospheres were counted and imaged using an inverted‐based reagent, TOX8 (Sigma). After incubation for 16 h, the fluorescence of the solutions was read at 590 nm (*λ*_ex_ = 560 nm). Viable mammospheres reduce the amount of the oxidized TOX8 (blue) and concurrently increase the amount of the fluorescent TOX8 intermediate (red), indicating the degree of mammosphere cytotoxicity caused by the test compound. Fluorescence values were normalized to DMSO‐containing or no DMSO‐containing controls and plotted as the concentration of test compound versus % mammospheres viability. IC_50_ values were interpolated from the resulting dose‐dependent curves. The reported IC_50_ values were the average of three independent experiments, each consisting of two replicates per concentration level (overall *n* = 4).

### Haemolysis (Human Red Blood Cells)

Fresh human red blood cells were obtained from Interstate Blood Bank, Inc., and thoroughly washed in PBS until the supernatant was clear. The peptide was serially diluted in PBS starting at a concentration of 200 µm. The final volume of peptide in each well was 50 µL. To each well, 50 µL of RBCs in PBS at 2 × 10^8^ cells/mL was added. As a positive lysis control, 1% triton was used. The mixtures were incubated at 37 °C for 1 h, after which they were centrifuged at 1000 × *g* for 5 min. After centrifugation, 10 µL of supernatant was transferred to 90 µL of ddH2O in a fresh 96‐well plate. The absorbance of released hemoglobin at 410 nm was recorded and the fractional haemolysis was calculated based on the 100% and 0% lysis controls.

### Sytox Green Assay to Measure Cytotoxicity against Hela Cells

Hela cells were grown to confluency in T‐75 flasks in complete DMEM with 10% FBS. The day prior to cytotoxicity experiments, cells were trypsinized, removed from the flask, and pelleted at 1300 rpm. The trypsin and media were discarded, and the cells were resuspended in complete DMEM. The cell count was obtained using a cell counter. The cells were then seeded at a density of 10 000 cells/well in a 96‐well tissue‐culture plate and incubated overnight. The next day, in a separate 96‐well plate, the peptide was serially diluted in complete DMEM (10% with FBS) and 0.1% sytox green starting at a concentration of 100 µm (1st), 50 µm (2nd) which was followed by 2:3 serial dilutions. The final volume of peptide in each well was 120 µL. To perform the cytotoxicity assay, media was removed from the wells and replaced with 100 µL peptide/DMEM/sytox green solutions. No peptide and 20 µm MelP5 were used as negative and positive controls, respectively. The plate was read for fluorescence every 5 min for an hour with an excitation wavelength of 504 nm and an emission wavelength of 523 nm. Cytotoxicity was calculated from the 100% and 0% lysis controls based on SYTOX green entry into the cells.

### ANTS/DPX Liposome Leakage Assay

Vesicle preparation: 5 mm ANTS and 12.5 mm DPX were entrapped in 0.1 µm diameter extruded vesicles (either POPC or POPC/POPG at a ratio of 3:1) with lipids. Gel filtration chromatography of Sephadex G‐100 was used to remove external free ANTS/DPX from LUVs with entrapped contents. LUVs were diluted to 0.5 mm and used to measure the leakage activity by the addition of aliquots of ACPs. Leakage was measured after 3 h incubation. 10% Triton was used as the positive control to measure the maximum leakage of the vesicle. Fluorescence emission spectra were recorded using excitation and emission wavelength of 350 and 510 nm for ANTS/DPX using a BioTek Synergy H1 Hybrid Multi‐Mode Reader.

### Tryptophan Fluorescent Binding Assay

50 µm ACP peptides and varied concentrations (concentrations: 0, 25, 50, 100, 150, 200, 250, 500, 1250, and 2500 µm) of POPC or POPC:POPG (3:1 ratio) large unilamellar vesicles were prepared in 10 mm phosphate buffer (pH 7.0). The solutions were measured after 1 h of incubation. Excitation was fixed at 280 nm (slit 9 nm) and emission was collected from 300 to 450 nm (slit 9 nm). The spectra were recorded using Cytation 5 Cell Imaging Multi‐Mode Reader from BioTek and were averaged by three scans.

### Molecular Dynamics Simulations and Analysis

Unbiased all‐atom MD simulations were performed and analyzed using GROMACS 2018.3 (www.gromacs.org),^[^ ^30^ ^]^ Hippo BETA (http://www.biowerkzeug.com),^[^ ^31^ ^]^ and VMD (http://www.ks.uiuc.edu/Research/vmd/).^[^ ^32^ ^]^

Extended peptide structures were generated using Hippo BETA. These initial structures were relaxed via 200 Monte Carlo steps, with water treated implicitly using a Generalized Born solvent. After relaxation, the peptides were placed in atomic detail peptide/lipid/water systems containing model membranes with 100 mm K and Cl ions using CHARMM‐GUI (http://www.charmm‐gui.org/).^[^ ^33^ ^]^ Protein folding simulations were equilibrated for 10 ns with applying position restraints to the peptide. For pore‐forming simulations, single peptides were allowed to fold onto the POPC bilayer for ≈600 ns; Once a stable surface state had been obtained, subsequently, the systems were multiplied 4 × 4 in the *x* and *y* (but not *z*) directions, resulting in a system with 16 peptides. When starting peptides from both sides of the membrane, the initial structure had one peptide in the upper and one in the lower leaflet. The large system was then constructed by multiplexing 3 × 3 to obtain an 18‐peptide simulation box. MD simulations were performed with GROMACS 2018.3 using the CHARMM36 force field,^[^ ^34^ ^]^ in conjunction with the TIP3P water model.^[^ ^35^ ^]^ Electrostatic interactions were computed using PME, and a cut‐off of 10 Å was used for van der Waals interactions. Bonds involving hydrogen atoms were constrained using LINCS.^[^ ^36^ ^]^ The integration time‐step was 2 fs and neighbor lists were updated every five steps. All simulations were performed in the NPT ensemble, without any restraints or biasing potentials. Water and the protein were each coupled separately to a heat bath with a time constant *τ*_T_ = 0.5 ps using velocity rescale temperature coupling. The atmospheric pressure of 1 bar was maintained using weak semi‐isotropic pressure coupling with compressibility *κ*_z_ = *κ*_xy_ = 4.6 × 10^−5^ bar^−1^ and time constant *τ*_P_ = 1 ps.

### Peptide Thermostability

Like other membrane‐active peptides, ACPs were stable against thermal denaturation even at 95 °C (Figure S9, Supporting Information),^[^ ^37^, ^38^ ^]^ allowing simulations to be carried out at elevated temperatures of 90 °C, which significantly enhances sampling. The authors had previously demonstrated that elevating the temperature does not change conformational equilibria or partitioning free energies of helical membrane‐active peptides, provided they were stable against thermal denaturation; however, the vast increase in sampling kinetics at high temperatures allows simulation of peptide folding, bilayer partitioning, and pore assembly.^[^ ^37^, ^38^, ^39^ ^]^

The key advantage of accelerated kinetics was the sufficient convergence of the peptide configurational equilibria, revealing equilibrium pore structures far from the initial starting conformation. In all simulations, the peptides inserted from their surface‐aligned states into transmembrane states and aggregated to form numerous pore aggregates. Peptide translocations between bilayer leaflets were frequently observed. The 30 µs simulations showed sufficient sampling of phase space for pore formation to occur. However, as big pores could take 20 µs to appear, it cannot be ruled out that simulation lengths in the 100–200 µs would provide even larger pore structures.

### Oligomer Population Analysis

In order to reveal the most populated pore assemblies during the simulations, a complete list of all oligomers was constructed for each trajectory frame. An oligomer of order *n* is any set of *n* peptides in mutual contact, with mutual contact defined as a minimum inter‐peptide heavy‐atom (N, C, O) distance of <3.5 Å. Frequently, this definition overcounted the oligomeric state due to numerous transient surface‐bound (S‐state) peptides that were only loosely attached to the transmembrane inserted peptides that made up the core of the oligomer. These S‐state peptides frequently changed position or drifted on and off the stable part of the pore. To focus the analysis on true longer‐lived TM pores, a cut‐off criterion of 75° was introduced for the tilt angle *τ* of the peptides. Any peptide with *τ* ≥ 75° was considered in the S‐state and removed from the oligomeric analysis. This strategy greatly reduced the noise in the oligomeric clustering algorithm by focusing on the true longer‐lived pore structures. Population plots of the occupation percentage of oligomer *n* multiplied by its number of peptides *n* were then constructed. These revealed how much peptide mass was concentrated in which oligomeric state during the simulation time.

### Permutational Cluster Analysis

All oligomers of the same order *n* were conformationally clustered using a clustering algorithm with a backbone RMSD similarity cutoff criterion of 4 Å. Since each oligomer could be made up of different peptides—or of the same peptides, but in a different order—the clustering compares one oligomer with all *n*! permutations of peptide arrangements of another oligomer. Permutations were generated using Heap's algorithm. The final RMSD value of the conformational similarity was considered the lowest RMSD value as obtained from the *n*! permutational comparisons. Clustering results were generally flat, indicating that structures were highly fleeting and dynamic.

### Transmembrane Flux

Water and ion flux through membrane pores was calculated by determining the total instantaneous flux through the whole bilayer patch. Two planes orthogonal to the membrane normal were considered at *z* = −7 Å and *z* = +7 Å, with all transition events that cross the planes counted. The flux was then obtained by dividing the transition counts by the area of the membrane patch and the elapsed time for each trajectory frame. Curves were subsequently smoothed by averaging over 1000 frames.

### Preparation and Characterization of Peptide Nanoparticles

In a typical preparation, 0.1 mL of 5 mg mL^−1^ L‐EEK peptide in methanol was mixed and sonicated with 1 mL of 25 mg mL^−1^ PEG‐PLGA in acetonitrile. The mixture was then added into 15 mL of 25 mm Tris buffer (pH 8.0), and the solution was stirred with a magnetic stirring bar in a 50 mL glass beaker at 400 rpm for 15 min. Methanol and acetonitrile were then evaporated from the solution completely via nitrogen gas bombardment for 15 min and upon placing the sample solution in vacuum for 1 h. The nanoparticle solution was then filtered through cellulose acetate syringe filters (pore size 0.45 µm, Sartorius). The filtered L‐EEK nanoparticles were washed with a 30 kDa centrifugal filter tube (Amicon Ultra‐15 Centrifugal Filter Devices) and concentrated to a final volume of 1 mL. The collected nanoparticles were freshly prepared for the experiments.

### Transmission Electron Microscopy

A drop (10 µL) of the L‐EEK nanoparticles solution (0.5 mg mL^−1^) was deposited onto a glow‐discharged grid. Negative staining was performed with 1 wt% uranyl acetate for structural examination of L‐EEK nanoparticles at room temperature. Negatively stained samples were visualized using the FEI 120 kV Sphera microscope (FEI Tecnai F20).

### Quantification of L‐EEK in Nanoparticles

The L‐EEK peptide in nanoparticles was quantified by HPLC. The HPLC analysis was carried out in an Agilent Technologies Series 1100 apparatus (Waldbronn, Germany). The analytical column was an Ascentis Express C18 reversed‐phase column (Supelco, Bellefonte, PA, USA) with a particle size of 5 µm (25 cm × 4.6 mm). The column temperature was maintained at 25 °C during the quantification. The mobile phase consisted of phase A (0.1% TFA in acetonitrile) and phase B (0.1% TFA in distilled water). The samples were started with linear gradient elution from 40% to 80% of phase A over 25 min, 80% to 100% of phase A from 25 to 30 min, and kept constant for 10 min. Then, the eluent was reversed to the initial composition within 5 min and kept constant for 5 min. The wavelength of detection was set at 220 nm for L‐EEK and the flow rate was at 0.7 mL min^−1^.

### Measurement of L‐EEK Release Rate from Nanoparticles

L‐EEK release from nanoparticles was studied using a dialysis tube with 20k MWCO Slide‐A‐Lyzer MINI dialysis device (Rockford, IL, USA) in PBS (pH 7.4) and in 0.15 m acetate buffer solution (pH 5.0). The phosphate buffer and the acetate buffer solution contained 0.3% v/v acetic acid and 1.3% w/v sodium acetate. The sample was placed into a dialysis tube at 37 °C under gentle stirring. At predetermined time points (1, 4, 8, 12, 24, 48, 72, and 96 h), the nanoparticle samples were collected and analyzed for L‐EEK content using HPLC.

### Haemolysis (Fresh Murine Red Blood Cells)

Fresh murine red blood cells were drawn from BALB/c nude mice and thoroughly washed in PBS until the supernatant was clear. L‐EEK and L‐EEK NPs were serially diluted in PBS starting at a concentration of 200 µm. Serial dilution of control NPs was based on the amount of polymer compared with EEK NPs. The final volume of peptide in each well was 50 µL. To each well, 50 µL of RBCs in PBS at 2 × 10^8^ cells/mL was added. As a positive lysis control, 1% triton was used. The mixtures were incubated at 37 °C for 1 h, after which they were centrifuged at 1000 × *g* for 5 min. After centrifugation, 10 µL of supernatant was transferred to 90 µL of distilled water in a fresh 96‐well plate. The absorbance of released hemoglobin at 410 nm was recorded and the fractional haemolysis was calculated based on the 100% and 0% lysis controls.

### Cell Viability and Cytotoxicity Assays

The colorimetric Cell Counting Kit‐8 (CCK‐8) assay was used to determine the cell viability in cell proliferation and cytotoxicity of ACPs and conventional anticancer drugs. Briefly, 1 × 10^4^ cells were seeded in each well of a 96‐well microplate. Free peptides or nanoparticles containing various concentrations of peptides (0, 0.1, 0.2, 0.4, 0.8, 1.6, 3.1, 6.3, 12.5, 25, 50, and 100 µm) were added to the cells and incubated for 72 h at 37 °C in a humidified atmosphere containing 5% CO_2_. To each well of the plate was then added 10 µL of CCK‐8 solution and incubated for another 4 h. The absorbance of the solutions in each well was measured at 460 nm. IC_50_ values were interpolated from the resulting dose‐dependent curves. The reported IC_50_ values were the average of two independent experiments, each consisting of six replicates per concentration level (overall *n* = 3).

### Examining of L‐EEK Nanoparticle Cellular Uptake to Breast Cancer Cell Lines by Confocal Microscopy

Fluorophore‐conjugated L‐EEK was prepared by incubating L‐EEK with Alexa Fluor 647 NHS ester at a 10 to 1 molar ratio in methanol for 72 h. Following the conjugation, 0.1 mL of 5 mg mL^−1^ dye‐labeled L‐EEK peptide in methanol was mixed with 1 mL of 25 mg mL^−1^ PEG‐PLGA in acetonitrile. The mixture was then added into 15 mL of 25 mm Tris buffer (pH 8.0), and the solution was stirred with a magnetic stirring bar in a 50 mL glass beaker at 400 rpm for 15 min. Methanol and acetonitrile were then evaporated from the solution completely via nitrogen gas bombardment for 15 min and upon placing the sample solution in vacuum for 1 h. The nanoparticle solution was then filtered through cellulose acetate syringe filters (pore size 0.45 µm, Sartorius). The filtered L‐EEK nanoparticles were washed with a 100 kDa centrifugal filter tube (Amicon Ultra‐15 Centrifugal Filter Devices) and concentrated to a final volume of 1 mL. The collected nanoparticles were freshly prepared for the experiments. To observe the cellular uptake between cells and L‐EEK nanoparticles, fluorescent L‐EEK nanoparticles suspended in PBS were incubated with four breast cancer cell lines (6 × 10^4^ cells/well). Following 2 h of incubation in confocal dishes (covered‐glass‐bottom dish, SPL 200350), cells were washed three times with PBS to remove unbound free peptides and peptide nanoparticles. The resulting cells were stained with 10 µg mL^−1^ DAPI, fixed with 4% paraformaldehyde, and examined using a confocal fluorescence microscope (Zeiss LSM 880 with Airyscan).

### Evaluation of the L‐Form EEK Peptide Nanoparticles against Triple‐Negative MDA‐MB‐231 Tumor Growth in Mice

The experimental protocol was approved by the Academia Sinica Institutional Animal Care and Utilization Committee, Academia Sinica, Taipei, Taiwan (#15‐10‐868). BALB/c nude mice were inoculated with MDA‐MB‐231 tumor cells (4 × 10^6^ cells per mouse) subcutaneously on the right flank. The mice were randomly divided into two groups at 11 days post‐tumor inoculation. Mice were treated with control nanoparticles (without L‐EEK peptide) and peptide nanoparticles with 10 mg kg^−1^ of L‐EEK peptides by intravenous injection administration. During the treatment period tumor volume and body weight were measured three times per week. The survival endpoint was set while the tumor volume reached 1000 mm^3^. The survival curves of individual groups were compared by a log‐rank (Mantel–Cox) test.

## Conflict of Interest

The authors declare no conflict of interest.

## Author Contributions

C.H.C. and Y.‐H.L. contributed equally to this work. C.H.C., K.S., C.‐M.J.H., and M.B.U. designed the study. C.H.C. and M.B.U. designed the peptide library. C.‐M.J.H. and Y.‐H.L. designed the nanoparticle and in vivo experiments. C.H.C. performed most of the biophysical and in vitro experiments of the peptides. C.H.C. and A.E. cultured the cells for peptide library screening and performed the apoptosis and necroptosis assay. J.G. and W.C.W. performed haemolysis assay and SYTOX green assay. Y.‐H.L. and C.H.C. made the nanoparticles. Y.‐H.L. characterized the nanoparticles. Y.‐H.L. and Z.‐S.F. performed the cell viability of L‐EEK, L‐EEK NPs, and control NPs treatment against breast cancer cells. Y.‐H.L. and L.C.‐W.L. performed and analyzed the mouse model of triple‐negative breast cancer. C.H.C., Y.‐H.L., K.S., C.‐M.J.H., and M.B.U. analyzed the experimental data. J.P.U. and M.B.U. performed and analyzed the molecular dynamics simulations. C.H.C., C.‐M.J.H., and M.B.U. wrote the paper with input from all authors.

## Supporting information

Supporting Information

## Data Availability

The data that support the findings of this study are available in the supplementary material of this article.
