# Supplementary material for: Integrated Design of a Membrane‐Lytic Peptide‐Based Intravenous Nanotherapeutic Suppresses Triple‐Negative Breast Cancer
Source: Adv Sci (Weinh). 2022 Mar 4;9(13):2105506. doi: 10.1002/advs.202105506 (PMC9069370; doi:10.1002/advs.202105506)
Supplement: Supplementary file 1 — Supporting Information [file ADVS-9-2105506-s001.pdf]

ADVANCED SCIENCE
Open Access

Supporting Information

for *Adv. Sci.*, DOI 10.1002/advs.202105506

Integrated Design of a Membrane-Lytic Peptide-Based Intravenous Nanotherapeutic Suppresses Triple-Negative Breast Cancer

Charles H. Chen, Yu-Han Liu, Arvin Eskandari, Jenisha Ghimire, Leon Chien-Wei Lin, Zih-Syun Fang, William C. Wimley, Jakob P. Ulmschneider, Kogularamanan Suntharalingam, Che-Ming Jack Hu* and Martin B. Ulmschneider**

Extended Data Section

Name	Sequence†	MW (g/mol)	Net Charge	Iso-electric Point	*ΔG_{interfacial}* (kcal/mol)	Hydrophobic Moment (<μH>)
DEE	GLLDLLELLLEAAGW	1625	-2	3.69	-2.31	4.94
EEE	GLLELLELLLEAAGW	1639	-2	3.85	-1.52	5.56
HEE	GLLHLLELLLEAAGW	1647	-1	5.26	-2.58	4.72
KEE	GLLKLLELLLEAAGW	1638	0	7.0	-2.55	4.74
DHE	GLLDLLHLLLEAAGW	1633	-1	5.17	-3.37	3.95
EHE	GLLELLHLLLEAAGW	1647	-1	5.26	-2.58	4.62
HHE	GLLHLLHLLLEAAGW	1655	0	7.96	-3.64	3.73
KHE	GLLKLLHLLLEAAGW	1646	1	10.12	-3.61	3.75
DKE	GLLDLLKLLLEAAGW	1624	0	6.92	-3.34	3.98
EKE	GLLELLKLLLEAAGW	1638	0	7.00	-2.55	4.65
HKE	GLLHLLKLLLEAAGW	1646	1	10.12	-3.61	3.76
KKE	GLLKLLKLLLEAAGW	1637	2	10.73	-3.58	3.78
DEH	GLLDLLELLLHAAGW	1633	-1	5.17	-3.37	3.94
EEH	GLLELLELLLHAAGW	1647	-1	5.26	-2.58	4.54
HEH	GLLHLLELLLHAAGW	1655	0	7.96	-3.64	3.76
KEH	GLLKLLELLLHAAGW	1646	1	10.12	-3.61	3.78
DHH	GLLDLLHLLLHAAGW	1641	0	7.96	-4.43	2.93
EHH	GLLELLHLLLHAAGW	1655	0	7.96	-3.64	3.58
HHH	GLLHLLHLLLHAAGW	1663	1	14.0	-4.7	2.73
KHH	GLLKLLHLLLHAAGW	1654	2	14.0	-4.67	2.75
DKH	GLLDLLKLLLHAAGW	1632	1	10.12	-4.4	2.96
EKH	GLLELLKLLLHAAGW	1646	1	10.12	-3.61	3.61
HKH	GLLHLLKLLLHAAGW	1654	2	14.0	-4.67	2.76
KKH	GLLKLLKLLLHAAGW	1645	3	14.0	-4.64	2.78
DEK	GLLDLLELLLKAAGW	1624	0	6.92	-3.34	3.97
EEK	GLLELLELLLKAAGW	1638	0	7.0	-2.55	4.57
HEK	GLLHLLELLLKAAGW	1646	1	10.12	-3.61	3.78
KEK	GLLKLLELLLKAAGW	1637	2	10.73	-3.58	3.8
DHK	GLLDLLHLLLKAAGW	1632	1	10.12	-4.4	2.96
EHK	GLLELLHLLLKAAGW	1646	1	10.12	-3.61	3.61
HHK	GLLHLLHLLLKAAGW	1654	2	14.0	-4.67	2.76
KHK	GLLKLLHLLLKAAGW	1645	3	14.0	-4.64	2.78
DKK	GLLDLLKLLLKAAGW	1623	2	10.73	-4.37	2.99
EKK	GLLELLKLLLKAAGW	1637	2	10.73	-3.58	3.63
HKK	GLLHLLKLLLKAAGW	1645	3	14.0	-4.64	2.79
KKK	GLLKLLKLLLKAAGW	1636	4	14.0	-4.61	2.81

Extended Data Table 1 | Peptide sequences. All 36 leucine-rich peptide sequences were synthesized as L-form. $\Delta G_{interfacial}$ represent the binding free energy of peptide partition between water and membrane interface. $\Delta G_{interfacial}$ and hydrophobic moment were estimated using the Wimley-White hydrophobicity scale using the MPEx software. †N-terminus is free, C-terminus: -NH2.

Name	Sequence†	IC_{50} (µM)				
		HMLER	HMLER-shEcad	MCF-10A	U2OS	HEK293T
DEE	GLLDLLELLLEAAGW	5.55 ± 0.35	6.05 ± 2.76	8.40 ± 1.56	61.00 ± 1.41	9.91 ± 0.44
EEE	GLLELLELLLEAAGW	6.25 ± 1.77	10.55 ± 3.46	>200 ± 0	78.75 ± 5.30	50.00 ± 0
HEE	GLLHLLELLLEAAGW	6.50 ± 0.71	4.95 ± 0.49	58 ± 41	47.50 ± 11	10.25 ± 0.2
KEE	GLLKLLELLLEAAGW	3.75 ± 0.78	2.05 ± 0.21	>200 ± 0	49.38 ± 13.26	11.10 ± 1.84
DHE	GLLDLLHLLLEAAGW	3.75 ± 0.92	2.80 ± 0.14	22.65 ± 4.31	17.25 ± 1.06	11.05 ± 1.91
EHE	GLLELLHLLLEAAGW	3.90 ± 0.28	2.55 ± 0.07	107 ± 37	18.25 ± 1.06	13.00 ± 0.00
HHE	GLLHLLHLLLEAAGW	16.70 ± 2.26	10.75 ± 0.78	>200 ± 0	53.25 ± 5.30	18.35 ± 3.75
KHE	GLLKLLHLLLEAAGW	3.70 ± 0.14	2.92 ± 0.17	20.85 ± 5.87	53 ± 2.83	26.00 ± 2.83
DKE	GLLDLLKLLLEAAGW	2.10 ± 0	1.57 ± 0.33	4.75 ± 0.49	9.93 ± 0.25	7.80 ± 1.41
EKE	GLLELLKLLLEAAGW	1.80 ± 0.28	1.30 ± 0.14	8.80 ± 0	8.00 ± 0.71	5.15 ± 0.21
HKE	GLLHLLKLLLEAAGW	2.70 ± 0.28	1.90 ± 0.42	7.60 ± 0.42	15.95 ± 0.78	5.15 ± 0.64
KKE	GLLKLLKLLLEAAGW	2.05 ± 0.64	1.70 ± 0	2.90 ± 0.57	12.75 ± 1.20	4.40 ± 0.14
DEH	GLLDLLELLLHAAGW	5.35 ± 1.91	3.60 ± 0.42	20.25 ± 6.72	36.48 ± 3.92	10.50 ± 4.67
EEH	GLLELLELLLHAAGW	3.30 ± 0.14	3.60 ± 0.85	167 ± 47	19.60 ± 3.96	12.90 ± 0.14
HEH	GLLHLLELLLHAAGW	28.60 ± 10.75	10.95 ± 1.06	>200 ± 0	106 ± 14	8.80 ± 1.70
KEH	GLLKLLELLLHAAGW	3.30 ± 0.42	2.35 ± 0.07	20.00 ± 0	49.25 ± 5.30	5.65 ± 0.07
DHH	GLLDLLHLLLHAAGW	21.45 ± 2.47	11.90 ± 0.14	>200 ± 0	55.85 ± 18.88	6.40 ± 0.42
EHH	GLLELLHLLLHAAGW	25.35 ± 2.76	16.05 ± 3.18	150 ±71	117 ± 7	5.90 ± 0.14
HHH	GLLHLLHLLLHAAGW	21.10 ± 6.93	9.50 ± 0.99	79.00 ± 11.31	185 ± 7	30.60 ± 4.81
KHH	GLLKLLHLLLHAAGW	5.20 ± 0.71	4.45 ± 0.49	10.35 ± 0.49	39.75 ± 5.30	7.75 ± 0.78
DKH	GLLDLLKLLLHAAGW	3.45 ± 0.07	2.80 ± 0.28	7.05 ± 0.21	21.13 ± 3.01	6.15 ± 1.34
EKH	GLLELLKLLLHAAGW	2.75 ± 0.07	2.18 ± 0.31	7.90 ± 0.42	23.83 ± 0.81	6.65 ± 1.34
HKH	GLLHLLKLLLHAAGW	3.45 ± 0.21	3.10 ± 0.57	6.10 ± 0.00	18.88 ± 1.24	5.70 ± 0.99
KKH	GLLKLLKLLLHAAGW	2.40 ± 0.85	2.20 ± 0.42	1.75 ± 0.35	13.30 ± 0.99	4.00 ± 0.28
DEK	GLLDLLELLLKAAGW	1.14 ± 0.52	0.70 ± 0.07	145 ± 78	19.88 ± 2.65	6.80 ± 0.85
EEK	GLLELLELLLKAAGW	1.10 ± 0.14	1.08 ± 0.18	>200 ± 0	32.88 ± 4.07	8.25 ± 0.64
HEK	GLLHLLELLLKAAGW	2.35 ± 1.06	3.45 ± 2.19	29.80 ± 7.35	143.20 ± 80.33	6.95 ± 1.63
KEK	GLLKLLELLLKAAGW	1.60 ± 0.14	1.35 ± 0.07	1.55 ± 0.07	8.15 ± 0.49	3.20 ± 0.14
DHK	GLLDLLHLLLKAAGW	1.75 ±0.49	1.02 ± 0.12	5.05 ± 1.34	20.45 ± 2.05	7.30 ± 0.42
EHK	GLLELLHLLLKAAGW	1.35 ± 0.35	0.71 ± 0.13	3.60 ± 0.99	25.00 ± 0	5.35 ± 0.07
HHK	GLLHLLHLLLKAAGW	3.15 ± 0.07	1.75 ± 0.21	5.60 ± 0.14	12.65 ± 1.91	5.25 ± 1.48
KHK	GLLKLLHLLLKAAGW	2.59 ± 0.92	1.40 ± 0.14	1.81 ± 0.05	12.88 ± 1.24	3.55 ± 0.78
DKK	GLLDLLKLLLKAAGW	1.95 ± 0.35	1.20 ± 0.14	2.57 ± 0.52	10.40 ± 1.70	3.40 ± 0.28
EKK	GLLELLKLLLKAAGW	1.72 ± 0.21	1.19 ± 0.40	1.70 ± 0.11	12.20 ± 1.84	3.35 ± 0.49
HKK	GLLHLLKLLLKAAGW	3.40 ± 0.71	2.60 ± 0.42	2.18 ± 0.39	12.23 ± 0.11	9.50 ± 0.99
KKK	GLLKLLKLLLKAAGW	1.57 ± 0.24	1.35 ± 0.40	1.37 ± 0.24	9.65 ± 1.20	3.70 ± 0.14
d-DHK	GlldllhlllkaaGw	0.32 ± 0.07	0.23 ± 0.04	0.55 ± 0.06	1.24 ± 0.03	1.55 ± 0.37
d-DEK	GlldllelllkaaGw	0.44 ± 0.10	0.36 ± 0	0.57 ± 0.01	5.84 ± 0.04	3.28 ± 0.21
d-EEK	GllellelllkaaGw	0.29 ± 0.01	0.29 ± 0.01	1.07 ± 0.10	4.78 ± 0.02	2.82 ± 0.07
Doxorubicin	Doxorubicin	(2.5 ± 0.3) x10^{-3}	(3.0 ± 0.6) x10^{-3}	(6.4 ± 0.2) x10^{-1}	(1.5 ± 0.8) x10^{-2}	(1.1 ± 0.2) x10^{-4}
Salinomycin	Salinomycin	0.37 ± 0.08	0.92 ± 0.28	9.76 ± 2.28	-	0.41 ± 0.10

Extended Data Table 2 | Cytotoxicity of ACP and small molecule chemotherapeutics against different human cell lines. Half maximal inhibitory concentration, IC_{50}, for various cancerous (HMLER, HMLER-shEcad, U2SO) and non-cancerous (MCF-10A, HEK293T) of the combinatorial ACP library, three selected D-enantiomers (d-DHK, d-DEK, and d-EEK), and the two small molecule anticancer drugs doxorubicin and salinomycin. All data points are reported at least as duplicates. For D-enantiomers and small molecule anticancer drugs experiments were replicated six times. †N-terminus is free, C-terminus: $-NH_2$.

Name	Sequence†	Lipid Concentration-induced 50% Peptide Binding (L/P)	
		POPC Vesicle	3POPC/1POPG Vesicle
DEE	GLLDLLELLLEAAGW	0.63	0.38
EEE	GLLELLELLLEAAGW	1.33	4.50
HEE	GLLHLLELLLEAAGW	0.58	0.50
KEE	GLLKLLELLLEAAGW	0.20	0.20
DHE	GLLDLLHLLLEAAGW	1.00	0.72
EHE	GLLELLHLLLEAAGW	0.44	0.44
HHE	GLLHLLHLLLEAAGW	1.00	0.75
KHE	GLLKLLHLLLEAAGW	0.88	0.71
DKE	GLLDLLKLLLEAAGW	0.56	0.46
EKE	GLLELLKLLLEAAGW	2.75	1.63
HKE	GLLHLLKLLLEAAGW	0.75	0.25
KKE	GLLKLLKLLLEAAGW	0.48	0.44
DEH	GLLDLLELLLHAAGW	0.75	1.50
EEH	GLLELLELLLHAAGW	0.50	0.50
HEH	GLLHLLELLLHAAGW	0.88	3.50
KEH	GLLKLLELLLHAAGW	1.00	0.50
DHH	GLLDLLHLLLHAAGW	0.67	0.28
EHH	GLLELLHLLLHAAGW	0.94	0.69
HHH	GLLHLLHLLLHAAGW	0.46	0.82
KHH	GLLKLLHLLLHAAGW	3.50	0.94
DKH	GLLDLLKLLLHAAGW	10.00	0.46
EKH	GLLELLKLLLHAAGW	0.75	0.19
HKH	GLLHLLKLLLHAAGW	0.18	0.38
KKH	GLLKLLKLLLHAAGW	0.83	0.48
DEK	GLLDLLELLLKAAGW	1.50	1.67
EEK	GLLELLELLLKAAGW	0.48	4.50
HEK	GLLHLLELLLKAAGW	0.44	0.46
KEK	GLLKLLELLLKAAGW	0.47	0.50
DHK	GLLDLLHLLLKAAGW	0.20	0.38
EHK	GLLELLHLLLKAAGW	0.56	0.42
HHK	GLLHLLHLLLKAAGW	0.68	0.20
KHK	GLLKLLHLLLKAAGW	0.17	0.38
DKK	GLLDLLKLLLKAAGW	2.75	6.88
EKK	GLLELLKLLLKAAGW	1.50	0.63
HKK	GLLHLLKLLLKAAGW	0.18	0.14
KKK	GLLKLLKLLLKAAGW	1.25	1.70

Extended Data Table 3 | Tryptophan binding assay. The table shows lipid concentration-induced 50 % peptide binding onto liposome. 50 µM peptides were fixed and incubated with titrated lipid (POPC vesicle or 3POPC/1POPG vesicle) concentration (0, 12.5, 25, 50, 100, 250, 500, 1000, 2500, and 5000 µM) in phosphate buffered saline (1X, pH 7.4). The lipid concentration that causes 50 % peptide binding was determined using tryptophan fluorescent binding assay and the values are shown as lipid per peptide.

Name	Sequence†	Peptide Concentration-induced 50 % ANTS/DPX leakage at pH 7.4 (L/P)		Peptide Concentration-induced 50 % ANTS/DPX leakage at pH 4.8 (L/P)	
		POPC Vesicle	3POPC/1POPG Vesicle	POPC Vesicle	3POPC/1POPG Vesicle
DEE	GLLDLLELLLEAAGW	644	597	110	164
EEE	GLLELLELLLEAAGW	143	346	35	28
HEE	GLLHLLELLLEAAGW	876	792	100	122
KEE	GLLKLLELLLEAAGW	626	657	97	108
DHE	GLLDLLHLLLEAAGW	566	581	58	469
EHE	GLLELLHLLLEAAGW	952	706	75	384
HHE	GLLHLLHLLLEAAGW	842	411	143	113
KHE	GLLKLLHLLLEAAGW	981	188	183	156
DKE	GLLDLLKLLLEAAGW	747	411	21	469
EKE	GLLELLKLLLEAAGW	738	657	75	438
HKE	GLLHLLKLLLEAAGW	692	365	173	94
KKE	GLLKLLKLLLEAAGW	1133	346	143	131
DEH	GLLDLLELLLHAAGW	620	548	63	193
EEH	GLLELLELLLHAAGW	738	755	58	168
HEH	GLLHLLELLLHAAGW	995	505	173	24
KEH	GLLKLLELLLHAAGW	995	286	188	68
DHH	GLLDLLHLLLHAAGW	684	692	106	98
EHH	GLLELLHLLLHAAGW	1043	981	386	80
HHH	GLLHLLHLLLHAAGW	1133	469	173	50
KHH	GLLKLLHLLLHAAGW	1153	329	227	48
DKH	GLLDLLKLLLHAAGW	614	274	41	58
EKH	GLLELLKLLLHAAGW	608	365	55	88
HKH	GLLHLLKLLLHAAGW	1077	274	193	47
KKH	GLLKLLKLLLHAAGW	2119	227	164	49
DEK	GLLDLLELLLKAAGW	747	773	10	469
EEK	GLLELLELLLKAAGW	730	657	39	386
HEK	GLLHLLELLLKAAGW	755	386	97	411
KEK	GLLKLLELLLKAAGW	1173	438	110	126
DHK	GLLDLLHLLLKAAGW	747	469	47	183
EHK	GLLELLHLLLKAAGW	981	505	55	117
HHK	GLLHLLHLLLKAAGW	1133	160	149	111
KHK	GLLKLLHLLLKAAGW	1153	193	140	53
DKK	GLLDLLKLLLKAAGW	463	156	47	227
EKK	GLLELLKLLLKAAGW	1095	235	60	219
HKK	GLLHLLKLLLKAAGW	657	90	106	106
KKK	GLLKLLKLLLKAAGW	1369	115	156	81

Extended Data Table 4 | Peptide concentration-induced 50 % ANTS/DPX liposome leakage. 0.5 mM POPC and 3POPC/1POPG vesicles were fixed and incubated with titrated peptide concentration (0, 0.02, 0.04, 0.08, 0.16, 0.32, 0.64, 1.25, 2.5, 5, 10, and 20 μM) in each phosphate buffered saline (1X, pH 7.4) and hydrochloric acid-adjusted phoshate buffered saline (1X, pH 4.8). The values are shown as lipid per peptide.

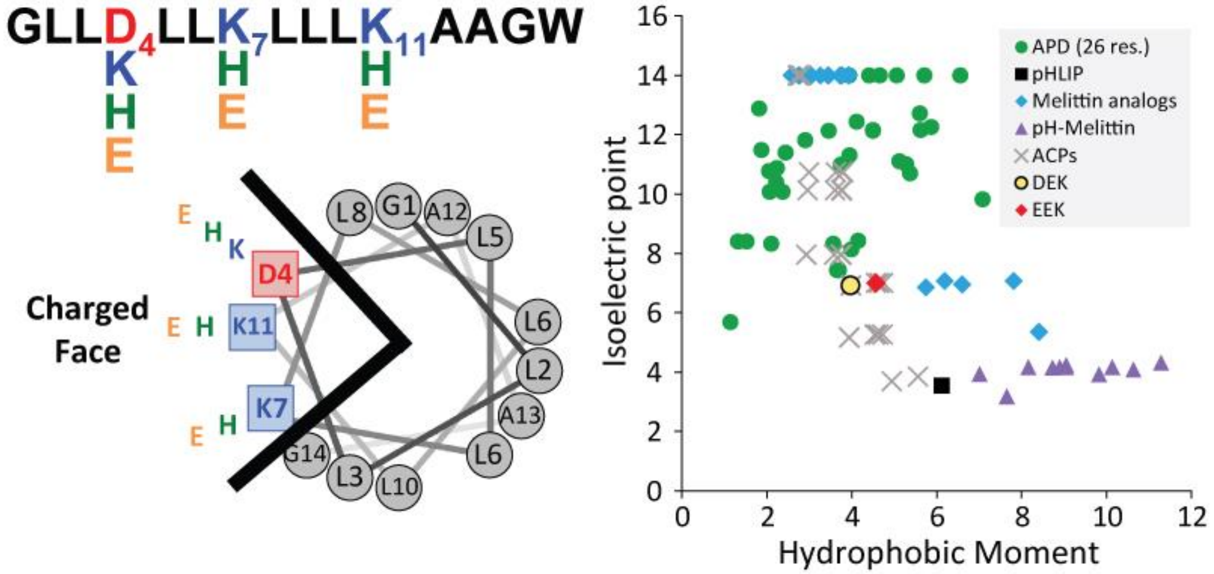

Extended Data Figure 1 | Anticancer peptide design and membrane conformation. Combinatorial 36-member leucine-rich membrane-active peptide library design. The template sequence is NH^{3+}-GLLxLLxLLLxAAGW-NH_2, where "x" can be the charged amino acids: aspartic acid, glutamic acid, histidine, or lysine. The helical wheel projection shows that all charged residues are located on the same polar face of the membrane-spanning helix; altering the charge distribution may affect the hydrophobic moment and isoelectric point.

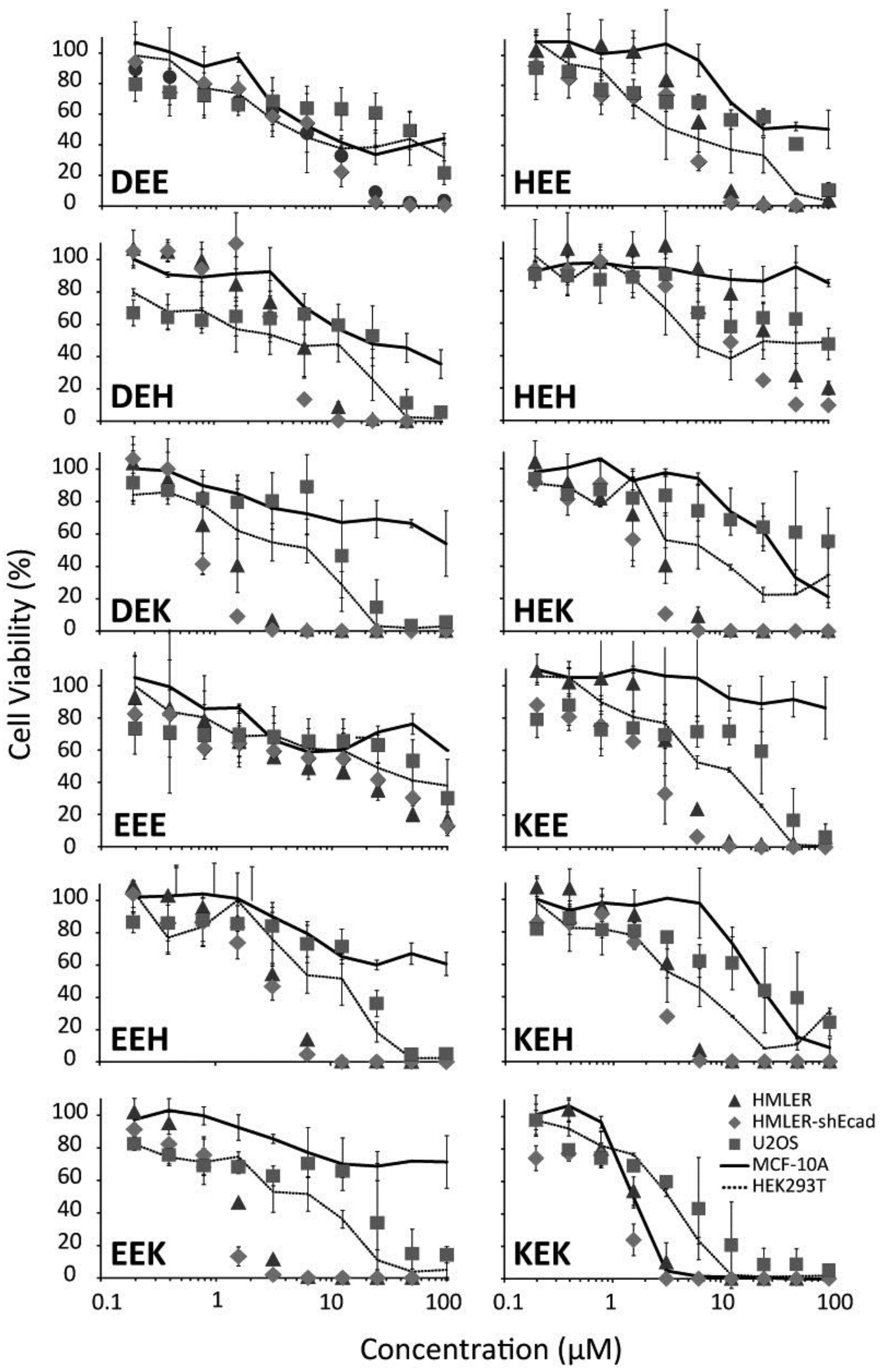

Extended Data Figure 2a | *In vitro* viability assay for cancerous (HMLER, HMLER-shEcad, U2OS) and non-cancerous (MCF-10A, HEK293T) cell lines in 2D cell culture models. a, Dose response of **x**E**y** peptides, where x = D, E, H, K and y = E, H, K.

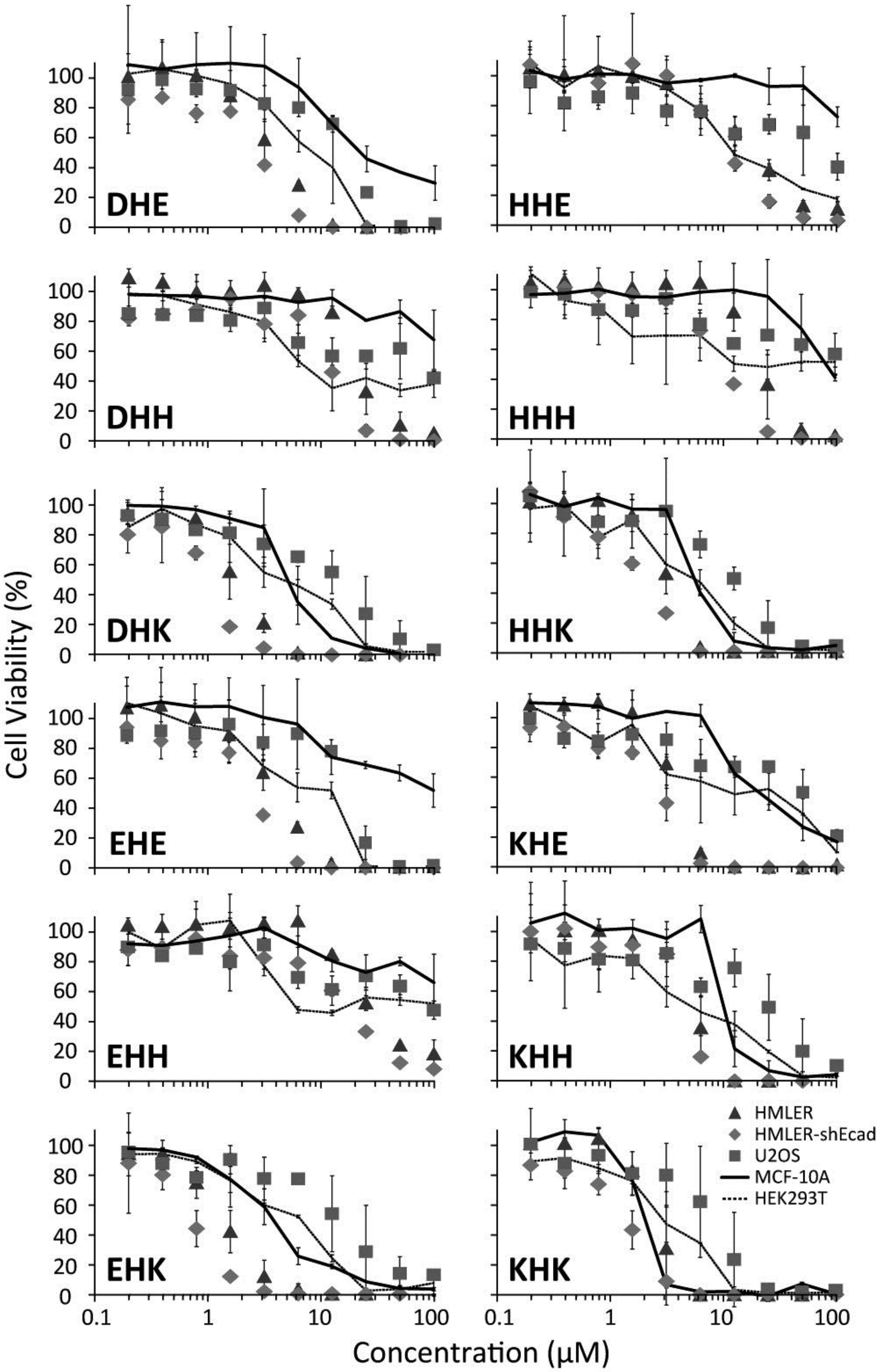

Extended Data Figure 2b | ***In vitro*** **viability assay for cancerous (HMLER, HMLER-shEcad, U2OS) and non-cancerous (MCF-10A, HEK293T) cell lines in 2D cell culture models. b,** Dose response of **x**H**y** peptides, where x = D, E, H, K and y = E, H, K.

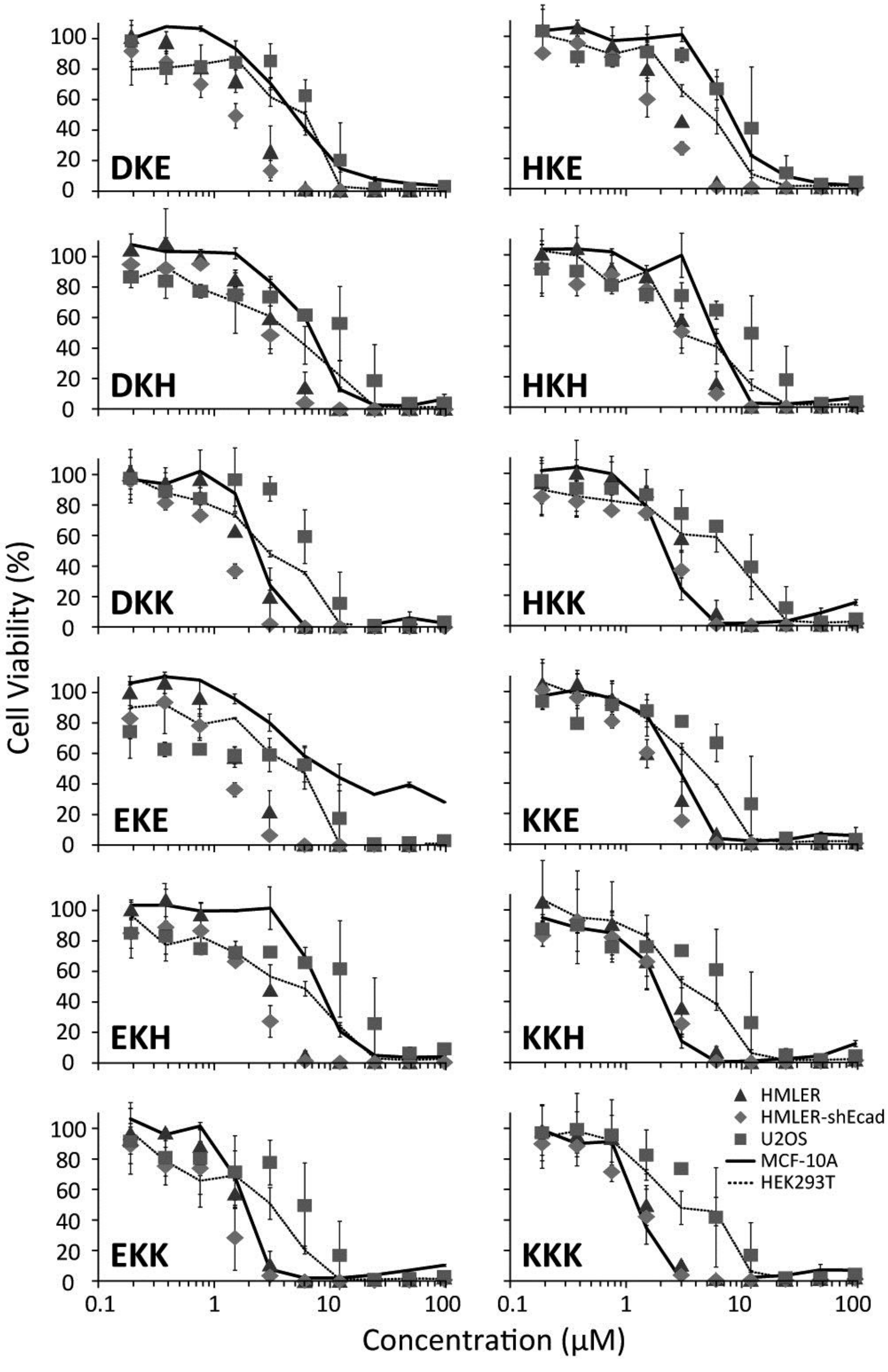

Extended Data Figure 2c | *In vitro* viability assay for cancerous (HMLER, HMLER-shEcad, U2OS) and non-cancerous (MCF-10A, HEK293T) cell lines in 2D cell culture models. c, Dose response of **x**K**y** peptides, where x = D, E, H, K and y = E, H, K.

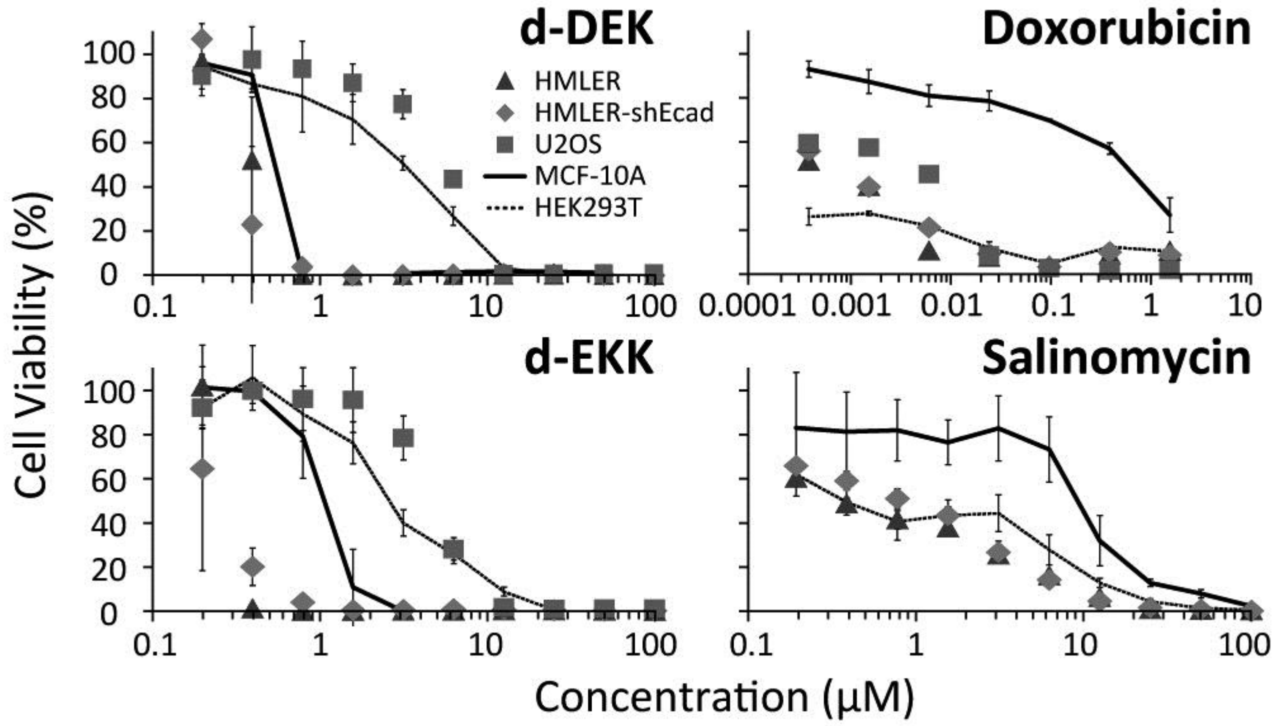

Extended Data Figure 3 | *In vitro* viability assay for cancerous (HMLER, HMLER-shEcad, U2OS) and non-cancerous (MCF-10A, HEK293T) cell lines in 2D cell culture models. d, Dose response of doxorubicin and salinomycin, three D-form anticancer peptides (d-DHK, d-DEK, d- EEK).

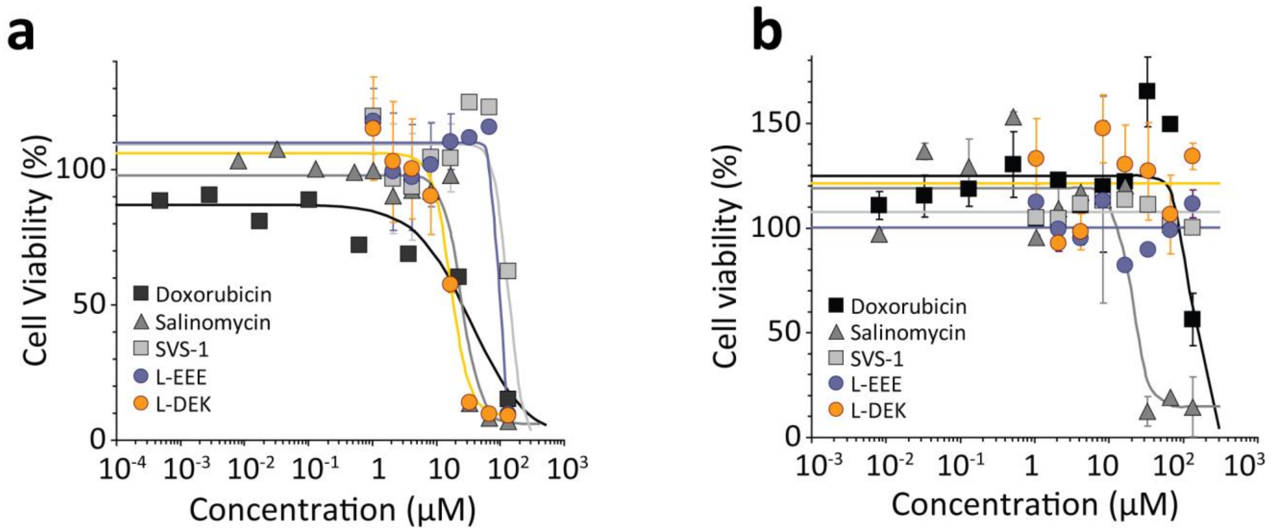

Extended Data Figure 4 | Comparison of compound activity against cancer stem cell spheroids. a, Dose-dependent cell viability of HMLER-shEcad tumourspheres treated with either doxorubicin, salinomycin, SVS-1, EEE, or DEK. **b,** Dose-dependent cell viability of MCF-10A mammospheres treated with the same compounds.

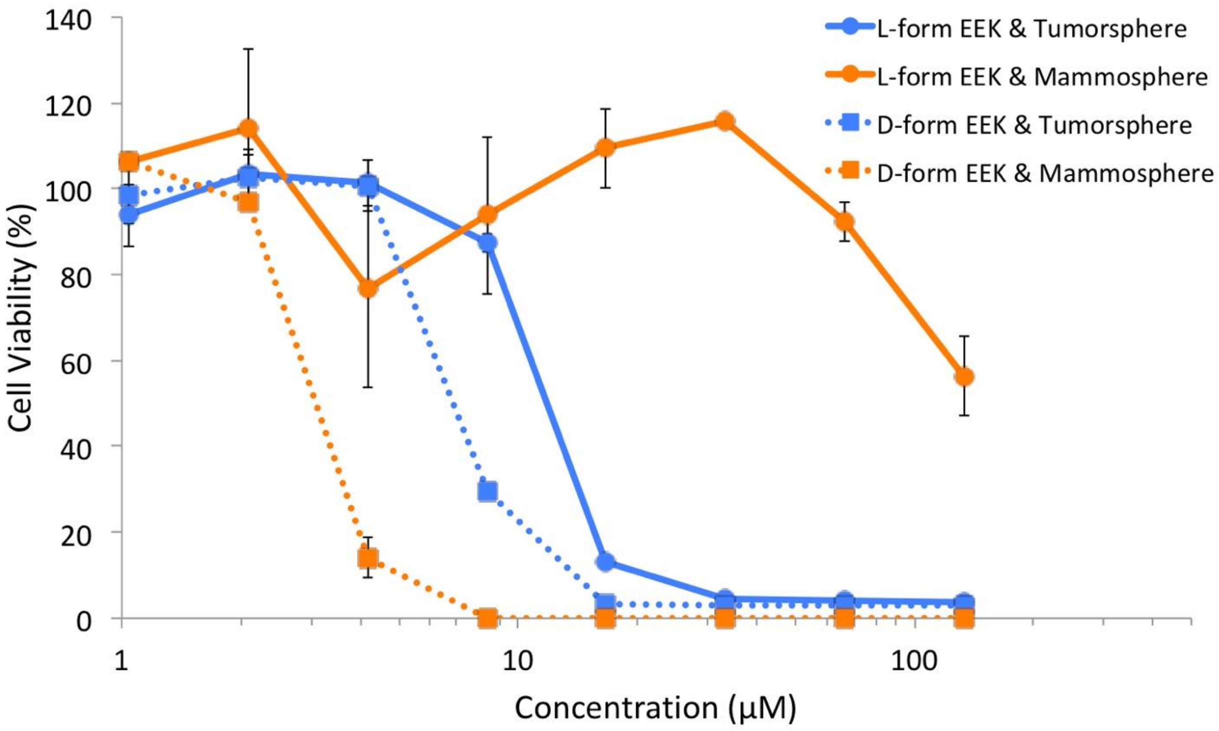

Extended Data Figure 5 | Cancer selectivity of EEK peptides against cancer stem cell spheroids. Dose-dependent cell viability of HMLER-shEcad tumourspheres and MCF-10A mammospheres treated with either L-form EEK peptide or D-form EEK peptide. All measurements were done in quadruplicate (n = 4).

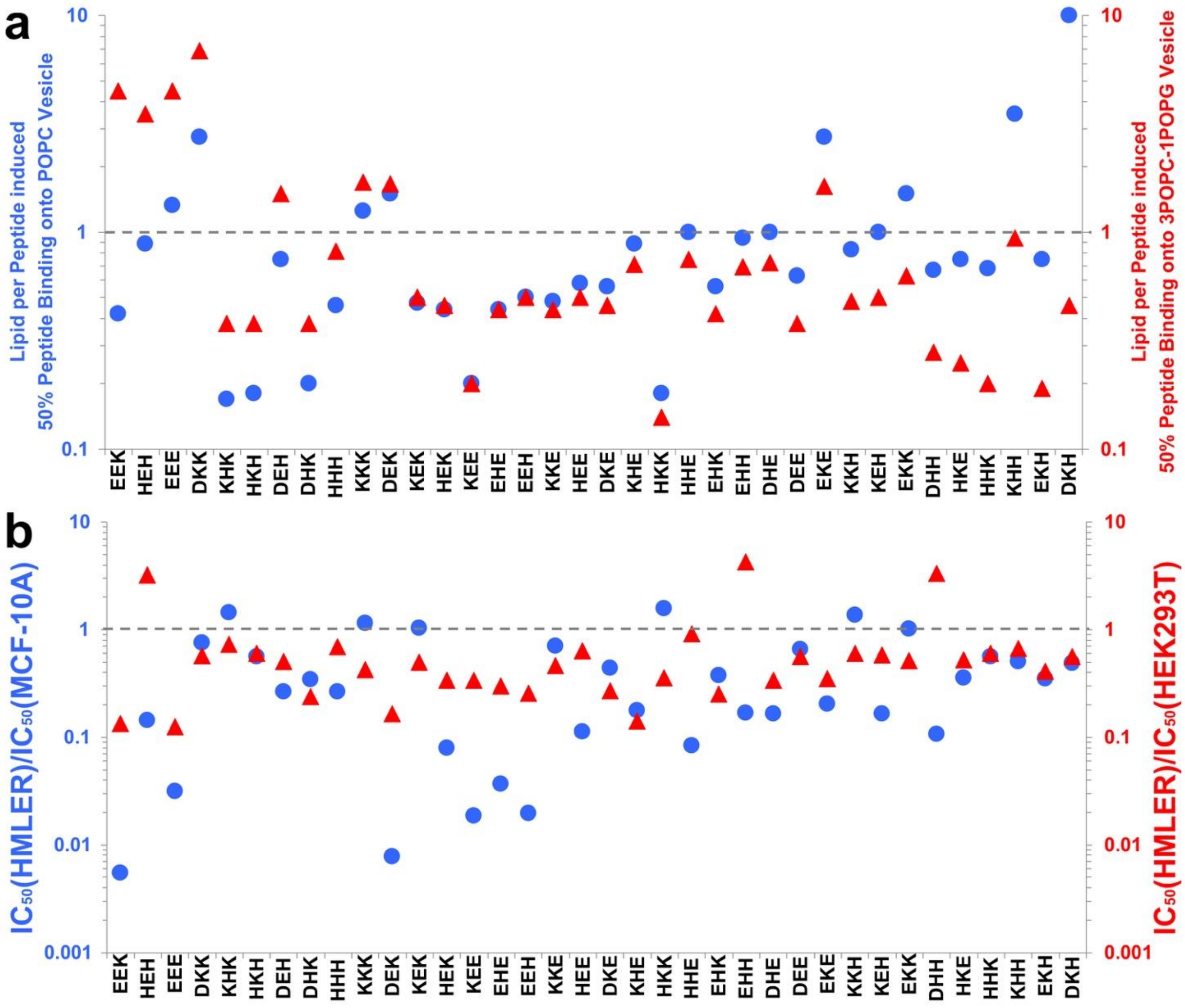

Extended Data Figure 6 | ACP binding to liposomes determined via tryptophan fluorescence. **a,** Lipid to ACP ratio at which 50% of each ACP binds to either a single lipid species POPC liposome (circles), or mixed lipid species POPC:POPG (ratio 3:1, squares) liposomes. The gray dashed line indicates a 1:1 peptide to lipid ratio. **b,** Cancer cell selectivity of the ACPs demonstrates the normalized selectivity. Blue colour indicates IC_{50}(HMLER)/IC_{50}(MCF-10A) and red colour indicates IC_{50}(HMLER)/IC_{50}(HEK293T). The gray dashed line indicates the normalized value is 1. Selectivity below 1 means the peptide is more favourable to kill cancerous HMLER cell rather than non-cancerous MCF-10A and HEK293T cells, and selectivity close to 1 or above 1 indicate cytotoxicity toward non-cancerous cells.

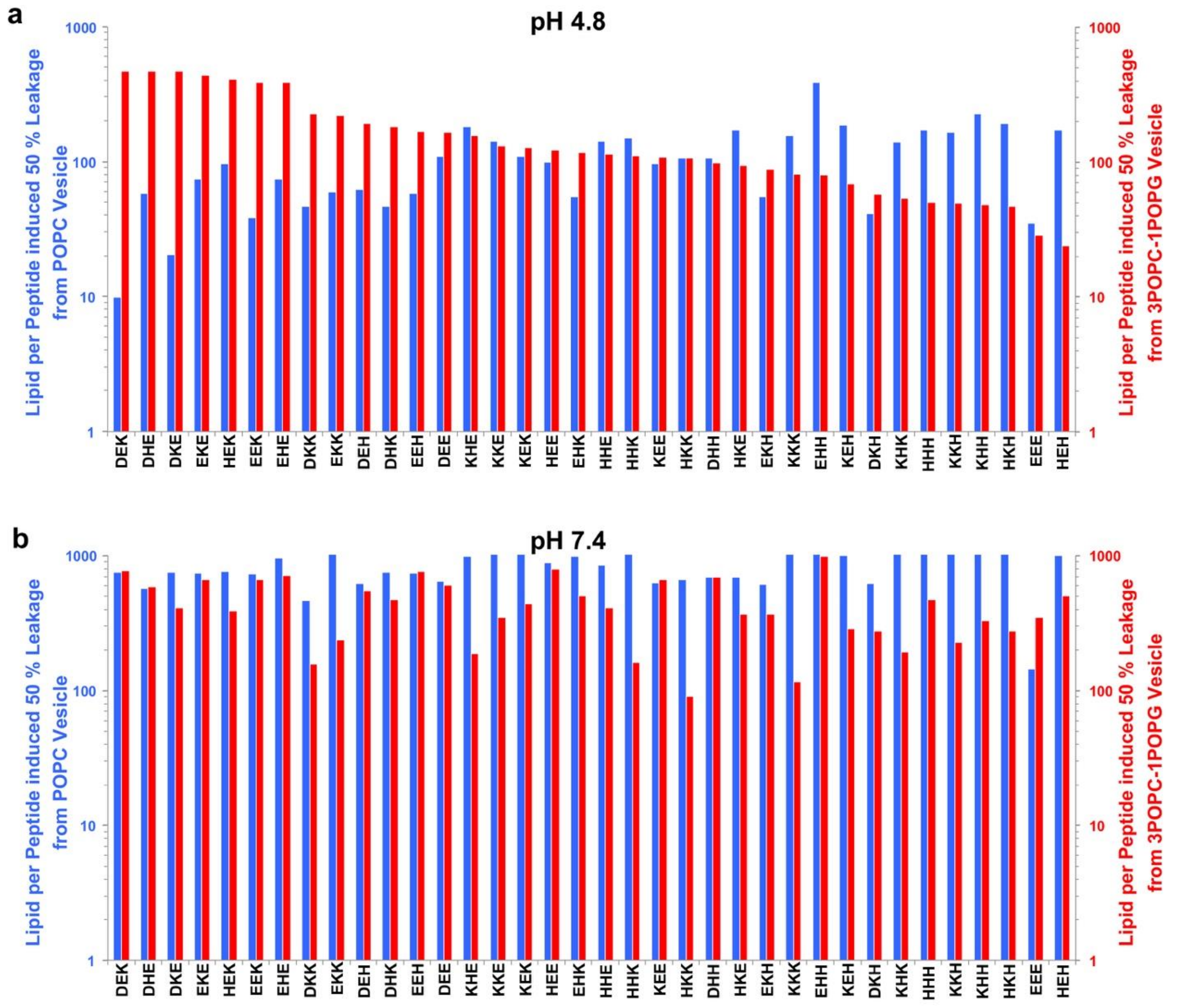

Extended Data Figure 7 | ACP concentration that induces 50 % ANTS/DPX leakage in POPC or POPC/POPG liposomes (1:3 ratio). ANTS/DPX leakage was measured for 0.5 mM liposome after incubation with ACP at concentrations of 0, 0.02, 0.04, 0.08, 0.16, 0.32, 0.64, 1.25, 2.5, 5, 10, and 20 µM in either **a,** hydrochloric acid-adjusted phoshate buffered saline (1X, pH 4.8) or **b,** phosphate buffered saline (1X, pH 7.4). High lipid to peptide ratios signify ACPs that are more potent at perforating the membrane.

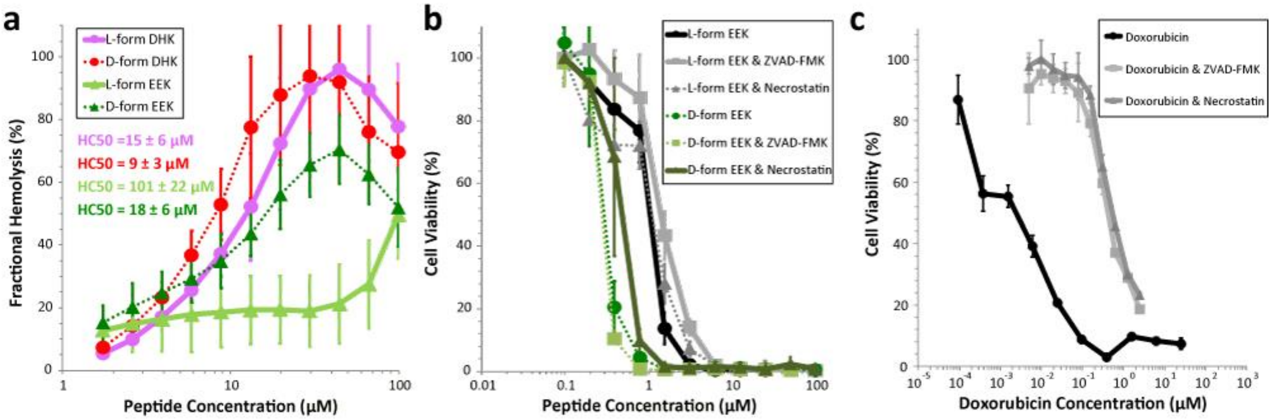

Extended Data Figure 8 | Mechanism of ACP anticancer activity. a, Haemolytic activity of DHK, d-DHK, EEK, and d-EEK against human red blood cells. **b,** HMLER-shEcad cell viability after treatment with EEK (IC_{50} = 1.2±0.1 µM), EEK together with 5 µM caspase inhibitor z-VAD-FMK (IC_{50} = 1.5±0.1 µM), EEK together with 20 µM necroptosis inhibitor necrostatin-1 (IC_{50} = 1.1±0.1 µM), d-EEK (IC_{50} = 0.3±0.1 µM), d-EEK and 5 µM z-VAD-FMK (IC_{50} = 0.3±0.1 µM), and d-EEK + 20 µM necrostatin-1 (IC_{50} = 0.5±0.1 µM). **c,** HMLER-shEcad cell viability after treatment with doxorubicin (IC_{50} = 3.1±0.4 nM), doxorubicin and 5 µM z-VAD-FMK (IC_{50} = 47.7±2.6 nM), and doxorubicin with 20 µM necrostatin-1 (IC_{50} = 63.1±4.2 nM).

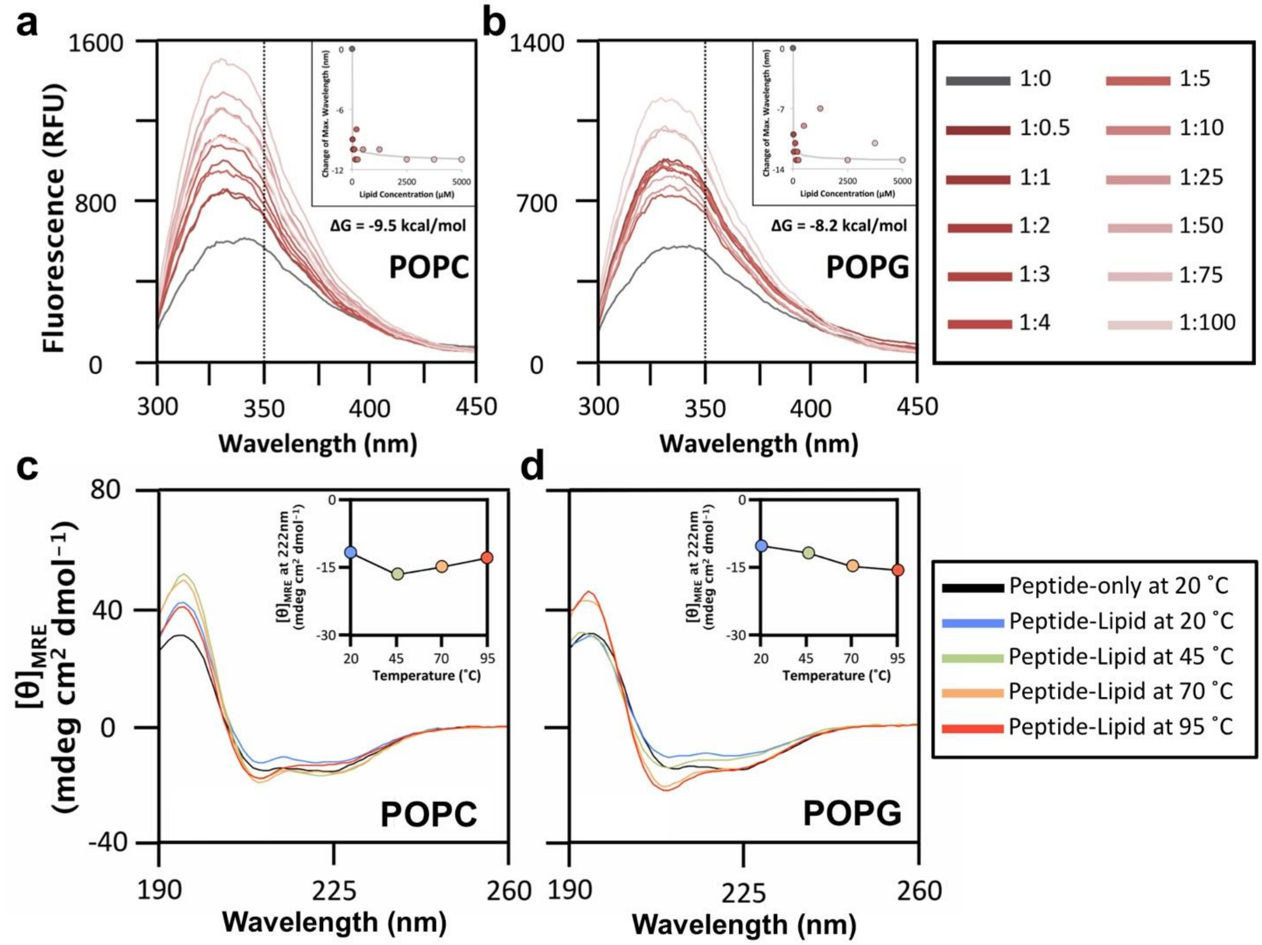

Extended Data Figure 9 | Peptide-lipid interactions. Conformational change of tryptophan fluorescence quenching of 50 μM DHK peptide with titrated **a,** POPC or **b,** POPG LUV concentration (0, 12.5, 25, 50, 100, 250, 500, 1000, 2500, and 5000 μM). Structural response of 50 μM DHK peptide with 600 μM of **c,** POPC or **d,** POPG LUV against elevated temperature (20, 45, 70, and 95 ˚C). The black solid line represents peptide-only in buffer at 20 ˚C. All the experiments were performed in 10 mM phosphate buffer, pH 7.0.

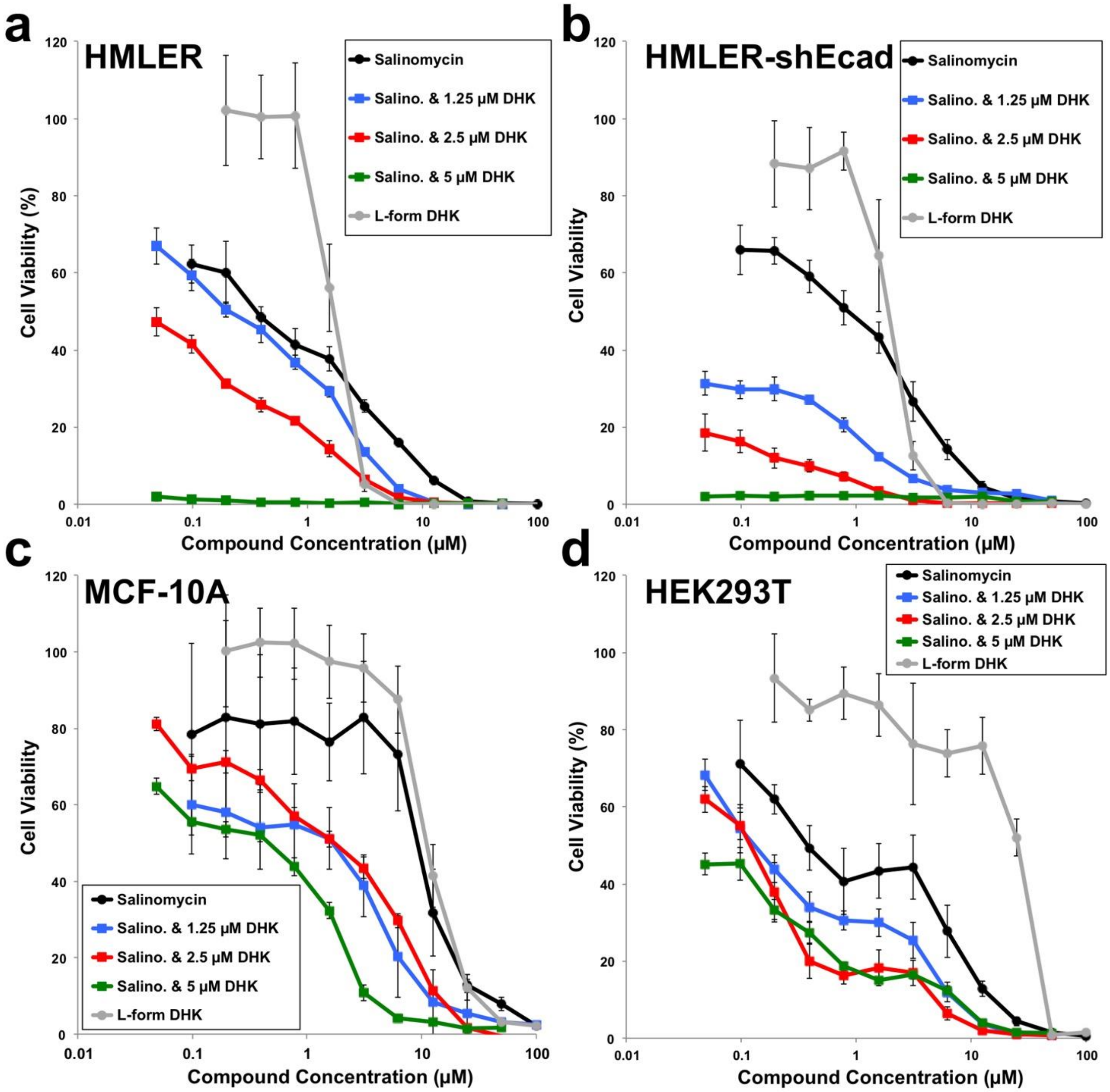

Extended Data Figure 10 | ACPs as small molecule chemotherapy enhancers. *In vitro* cytotoxicity dose-response measurement of salinomycin co-incubated with fixed concentrations (0, 1.25, 2.5 and 5 μM) of DHK against **a,** HMLER, **b,** HMLER-shEcad, **c,** MCF-10A, and **d,** HEK293T.

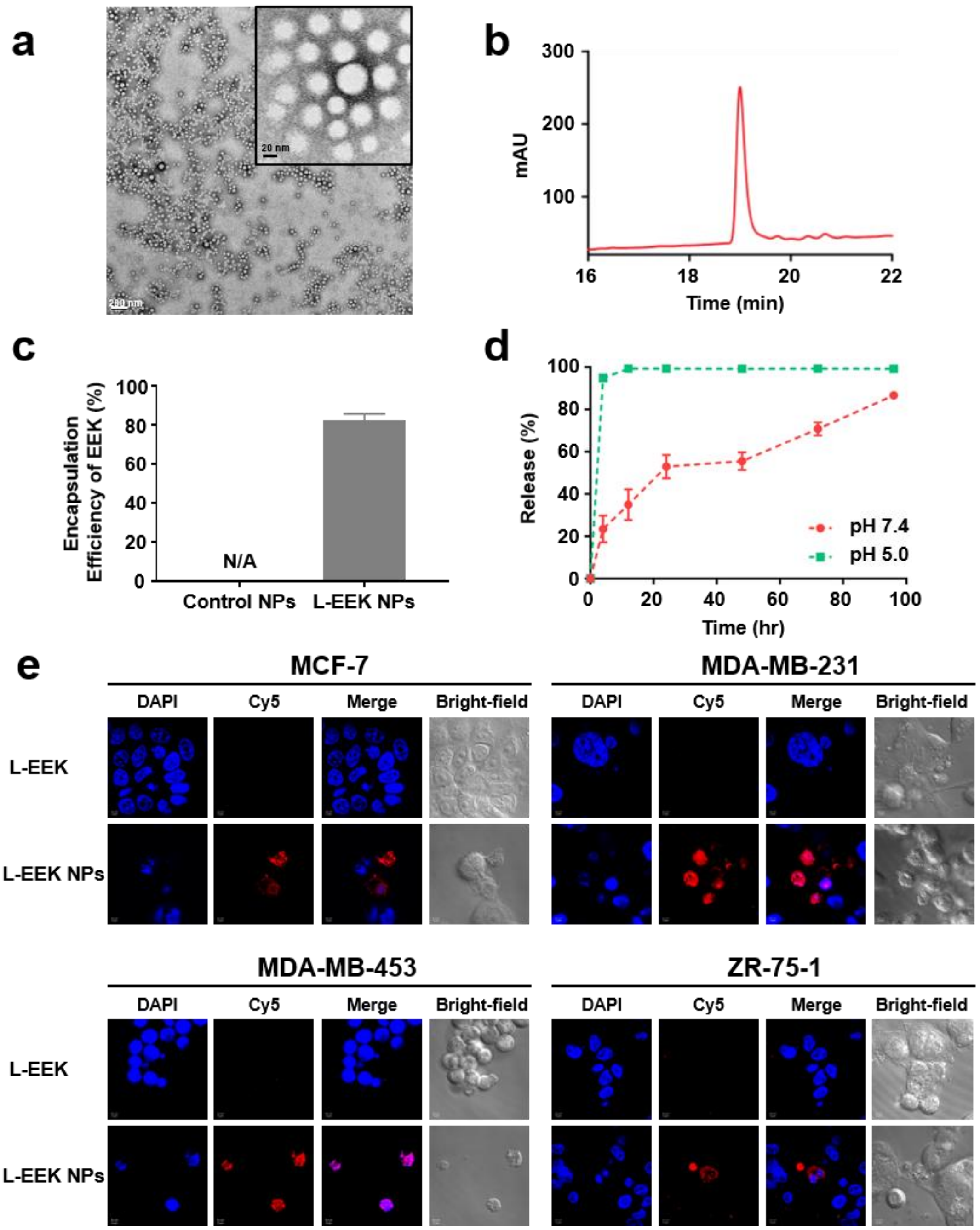

Extended Data Figure 11 | Physicochemical properties of L-EEK NPs. a, TEM images of control NPs. Scale bars = 200 nm and 20 nm in the inset. **b,** The chromatograms of L-EEK under HPLC. **c,** The encapsulation efficiency of L-EEK in L-EEK NPs. **d,** L-EEK release from nanoparticles at pH 7.4 and pH 5.0 at 37 ˚C. **e,** Cellular uptake of free and nanoparticle-encapsulated Alexa647-labelled L-EEK in breast cancer cell lines.

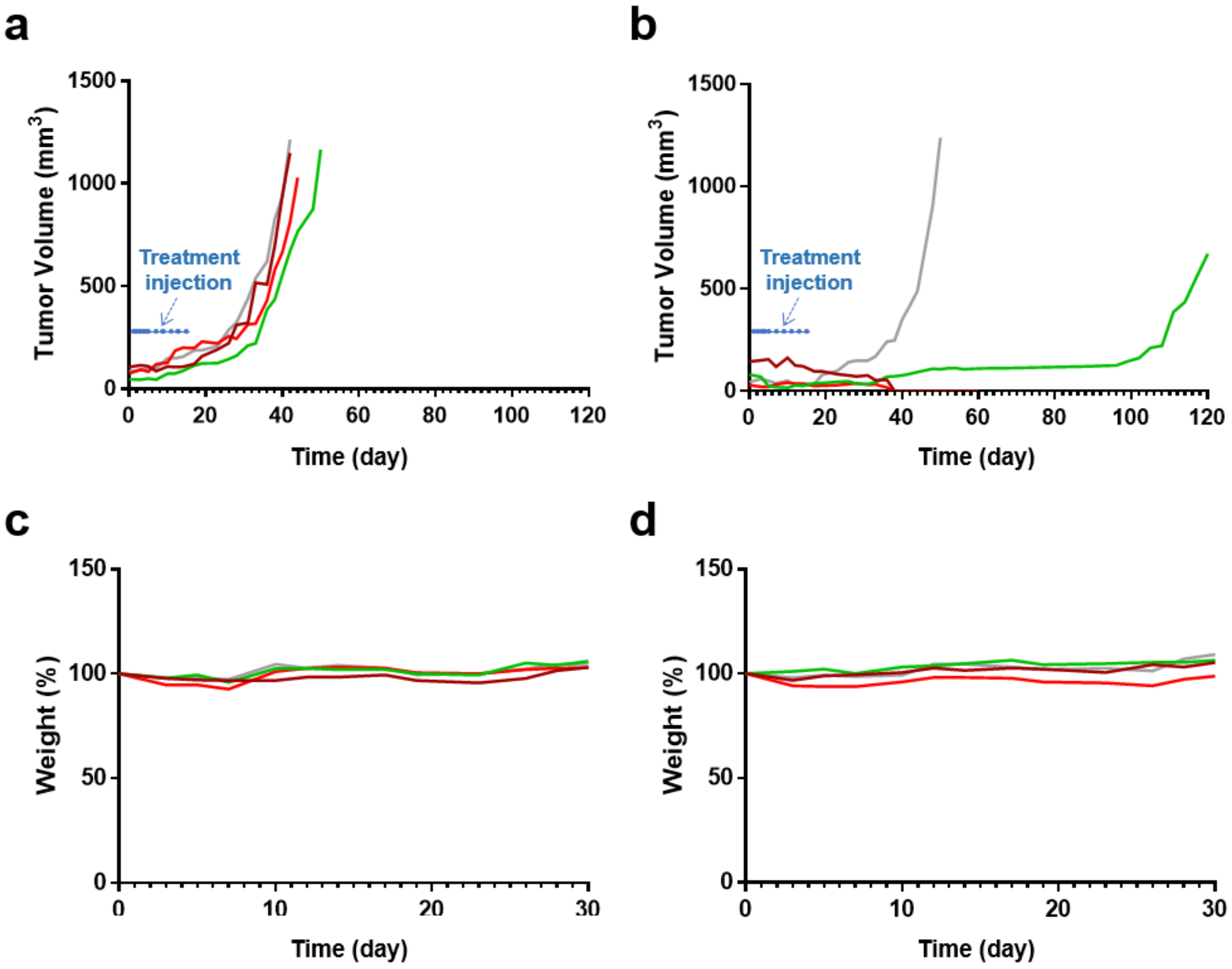

Extended Data Figure 12 | Potent anticancer efficacy with L-EEK NPs treatment. a, and c, Individual MDA-MB-231 tumour growth curves and body weight curves for mice treated with control nanoparticles (n = 4). b, and d, Individual MDA-MB-231 tumour growth curves and body weight curves for mice treated with L-EEK nanoparticles (n = 4).
